# Impaired autophagy increases susceptibility to endotoxin-induced chronic pancreatitis

**DOI:** 10.1038/s41419-020-03050-3

**Published:** 2020-10-21

**Authors:** L. Xia, Z. Xu, X. Zhou, F. Bergmann, N. Grabe, M. W. Büchler, J. P. Neoptolemos, T. Hackert, G. Kroemer, F. Fortunato

**Affiliations:** 1 Department of General, Visceral and Transplantation Surgery, University Hospital Heidelberg, Heidelberg, Germany; 2 Section Surgical Research, University Hospital Heidelberg, Heidelberg, Germany; 3 Institute of Pathology; University Hospital Heidelberg, Heidelberg, Germany; 4 National Center for Tumor Diseases (NCT), University Hospital Heidelberg, Heidelberg, Germany; 5 Equipe labellisée par la Ligue contre le cancer, Université de Paris, Sorbonne Université, INSERM U1138, Centre de Recherche des Cordeliers, Paris, France; 6 Metabolomics and Cell Biology Platforms, Institut Gustave Roussy, Villejuif, France; 7 Pôle de Biologie, Hôpital Européen Georges Pompidou, AP-HP, Paris, France; 8 Suzhou Institute for Systems Medicine, Chinese Academy of Medical Sciences, Suzhou, China; 9 Karolinska Institute, Department of Women’s and Children’s Health, Karolinska University Hospital, Stockholm, Sweden

**Keywords:** Acute pancreatitis, Chronic pancreatitis

## Abstract

Chronic pancreatitis (CP) is associated with elevated plasma levels of bacterial lipopolysaccharide (LPS) and we have demonstrated reduced acinar cell autophagy in human CP tissue. Therefore, we investigated the role of autophagy in experimental endotoxin-induced pancreatic injury and aimed to identify LPS in human CP tissue. Pancreatic Atg7-deficient mice were injected with a single sub-lethal dose of LPS. Expression of autophagy, apoptosis, necroptosis, and inflammatory markers was determined 3 and 24 h later utilizing immunoblotting and immunofluorescence. The presence of LPS in pancreatic tissue from mice and from patients and healthy controls was determined using immunohistochemistry, immunoblots, and chromogenic assay. Mice lacking pancreatic autophagy exhibited local signs of inflammation and were particularly sensitive to the toxic effect of LPS injection as compared to control mice. In response to LPS, *Atg*7^Δpan^ mice exhibited enhanced vacuolization of pancreatic acinar cells, increase in TLR4 expression coupled to enhanced expression of NF-κΒ, JNK, and pro-inflammatory cytokines by acinar cells and enhanced infiltration by myeloid cells (but not *Atg*7^F/F^ controls). Cell death was enhanced in *Atg*7^Δpan^ pancreata, but only necroptosis and trypsin activation was further amplified following LPS injection along with elevated pancreatic LPS. The presence of LPS was identified in the pancreata from all 14 CP patients examined but was absent in the pancreata from all 10 normal controls. Altogether, these results support a potential role for metabolic endotoxemia in the pathogenesis of CP. Moreover, the evidence also supports the notion that autophagy plays a major cytoprotective and anti-inflammatory role in the pancreas, and blunting metabolic endotoxemia-induced CP.

## Introduction

Chronic pancreatitis (CP) is a progressive fibro-inflammatory disease of the pancreas in which both exocrine and endocrine components are irreversibly damaged, eventually compromising the function of the organ^1,2^. CP is characterized by excessive apoptotic and necroptotic death of parenchymal cells, recruitment of inflammatory leukocytes, and progressive fibrogenesis^3–5^.

We have shown that human pancreatitis is associated with disabled autophagic ATG7 and ATG5, as well as a reduction of LAMP2 protein expression, impairing acinar cell autophagy signaling^4–6^. In addition, we and others have shown that genetic intervention leading to the local removal of essential autophagy genes such as autophagy-related 5 and 7, *Atg5, Atg*7, or lysosomal-associated membrane protein-2 (*Lamp2*) promotes the development of pancreatitis in rodents^3,5–9^.

Autophagy is an evolutionarily conserved process in which cytoplasmic material is engulfed in autophagosomes and then delivered to lysosomes for digestion and recycling of the luminal content^10,11^. Basal autophagy levels are particularly high in acinar cells of the exocrine pancreas, which synthesize digestive enzymes^12^.

Autophagy has cytoprotective functions in multiple cell types by virtue of its capacity to digest macromolecules to energy-rich metabolites^13^, to mediate anti-inflammatory effects, and to eliminate potentially harmful structures including dysfunctional organelles and infectious pathogens^10,14^. In pancreatic acinar cells, autophagy can participate in the neutralization of deleterious activated zymogen granules, a phenomenon that is referred to as “zymophagy”^15^. Moreover, autophagy can capture intracellular bacteria or viruses in a process that is referred to as “xenophagy”^10,16–21^.

The gut microbiome has distinct features in patients with CP and also animal models of CP^22–24^. Jandhyala et al. showed that there were increased plasma bacterial lipopolysaccharide (LPS) levels in patients with CP. The higher levels of endotoxin were also associated with a relative abundance in LPS synthetic pathways of gut microflora in the patients with CP compared to controls^22^.

Sepsis is a life-threatening illness with extensive tissue damage and often lethal multi-organ failure caused by the deregulated inflammatory response to an infection, mostly by bacteria producing LPS, also called endotoxin^25–27^. Systemic inflammatory response syndrome (SIRS) and the compensatory anti-inflammatory response syndrome (CARS) are two opposite stages of the immune response during sepsis, in which a mixed anti-inflammatory response representing a temporary balance between decreasing SIRS and increasing CARS may also manifest^28^. Derailment of this balance in either direction can lead to septic shock and death.

LPS binds to the CD14/TLR4/MD2 receptor complex, inducing an inflammatory response. Metabolic endotoxemia refers to the state in which plasma LPS levels are elevated, regardless of the presence of obvious infection^29^.

In models of acute pancreatitis, the pancreatic acinar cells respond to endotoxin by producing inflammatory mediators, thus triggering local inflammation^30,31^. LPS can suppress late-stage of autophagy by reducing the expression of Lamp-2^6^. Pancreatic autophagy-deficient mice (*Atg7*^Δpan^) develop features of CP including vacuolization of acinar cells, tissue edema, fibrosis, apoptosis, and infiltration by inflammatory cells^5^. Here, we show that in response to LPS, *Atg*7^Δpan^ mice exhibited enhanced vacuolization of pancreatic acinar cells, enhanced infiltration by myeloid cells, exacerbated trypsin activation and accumulation of LPS in pancreatic tissues, but not in autophagy-competent mice. We also demonstrate the presence of LPS in pancreatic tissue from patients with CP but not in healthy normal pancreatic tissue. Taken together these studies support a potential role for metabolic endotoxemia in the pathogenesis of CP and demonstrate an important role for autophagy in suppressing its development.

## Materials and methods

### Antibodies and reagents

Antibodies were selected according to proven functionality for formalin-fixed paraffin-embedded (FFPE) tissue sections and WB by the seller or by publication records. All antibodies used in this investigation are listed in the supplemental Material and Methods.

### Human subjects

The study was approved by the Ethics Committee of the University Medical Faculty of Heidelberg. All patients signed the consent form and were informed that their tissue will be used in research. Human pancreata were obtained from 17 CP patients (8 females, 9 males, median age 45 years), randomly collected from patients visiting the Department of Surgery of our University. Ten healthy donor pancreatic tissue (5 females, 5 males, median age 54 years) were obtained from patients who were free of pancreatic disease, through an organ donor program in which there were no candidates for pancreatic transplantation. Surgically removed tissue samples were immediately fixed in 4% buffered formalin or snap-frozen in liquid nitrogen and stored at −80 °C for protein extraction.

### Human pancreatic histopathology

FFPE pancreatic tissue was cut into 4-μm-thick sections and stained with hematoxylin and eosin (H&E). H&E staining tissue sections were checked by a single patho-logist for the severity of pancreatic parenchymal edema, inflammation, apoptosis, fibrosis, and acinar cell vacuolization in a blinded manner, as described before^5,6,32,33^.

Histopathological evaluation of the pancreatic tissue included: the severity of fibrosis, the severity of inflammation, the activity of inflammation, and the severity and activity of the perineural inflammation, as described^33^. The severity of fibrosis was determined by the modified evaluation described by Ammann and colleagues^32^, using a scoring system based on focal versus diffuse extension of intralobular and perilobular fibrosis. The severity and distribution of fibrosis in the investigated specimens were graded according to a scoring system shown in Table 1. Severity of fibrosis was determined by the use of an intralobular and perilobular fibrosis score of mild 0 (0–4), moderate I (5–9), or severe II (10–12) fibrosis.

**Table 1:** Histopathological evaluation of human chronic pancreatitis specimens.

Control *n* = 10	Chronic pancreatitis *n* = 14			
**Intralobular fibrosis**		**Intralobular fibrosis**		
Absent	Absent	Mild	Moderate	Severe
0	1	2	4	7
**Perilobular fibrosis**		**Perilobular fibrosis**		
Mild	Absent	Mild	Moderate	Severe
5	0	1	2	11
**Inflammation**		**Inflammation**		
Absent	Absent	Mild	Moderate	Severe
0	0	12	2	0
**Inflammatory activity**		**Inflammatory activity**		
Absent	Absent	Mild	Moderate	Severe
0	5	9	0	0
**PanIN**		**PanIN**		
Mild	Absent	Mild	Moderate/Severe	
2	7	5	2	
**Fat Necrosis**		**Fat Necrosis**		
Absent	Absent	Mild	Moderate/Severe	
0	12	2	0	
**Acinar-to-Ductal metaplasia**		**Acinar-to-Ductal metaplasia**		
Absent	Absent	Mild	Moderate/Severe	
0	3	9	2	

Tissue scores were determined according to the previously described tissue scoring system^4^.

The severity of inflammation was scored as absent (0), mild (I), moderate (II), or severe (III), based on the determination of the overall accumulation of inflammatory cells (lymphocytes, plasma cells, and macrophages). The activity of inflammation was based on the presence and density of neutrophil granulocytes representing the early inflammatory response. These were scored as absent (0), mild (I) or moderate to severe (II). Overall severity of inflammation was scored as absent (0), mild (0–1), moderate (2–3), or severe (4–6). Acinar-to-ductal metaplasia (ADM) was scored according to the tissue frequency, with scores of absent (0), mild (I), or moderate to severe (II). Pancreatic tissues from mice were scored very similarly.

### Animals

Atg7 flox/flox (Atg7^F/F^) mice, containing floxed alleles for the autophagy gene *Atg7*, were provided by Masaaki Komatzu and Erwin Tschachler^34,35^. Atg7^F/F^ mice were crossed with Ptf1a/p48-cre mice with p48 promoter-driven *cre* recombinase to generate p48-Cre-Atg7^F/F^ mice (Atg7^Δpan^ mice), resulting in a pancreas-specific auto-phagy defect, as previously described^5,36^. Hemizygote mutant males LAMP-2^y/−^ mice were provided by Paul Saftig and were obtained from crossed wild-type males (Lamp2^+/+^) with heterozygote females (Lamp2^+/−^), resulting in the loss of LAMP2^y/−^ in all cells of the mouse. The mice were housed under standard condition and maintained with a C57LB/6 background. All animal studies were approved by the Institutional Animal Care and Use Committee of Heidelberg University, and followed the Federal Presiding Board guidelines for Animal Care, Karlsruhe, Germany.

### Experimental design

All studies were performed in 12-week-old male mice. Each strain of mice were divided into six groups with 4–6 animals for each group: *Atg7*^F/F^ and *Atg7*^Δpan^ as well as *Lamp2*^*+/+*^ and *Lamp2*^*y/*−^ mice with or without LPS, were anesthetized 3 or 24 h after LPS injection. Gram negative bacterial LPS (Escherichia coli O26:B6; 5 mg/kg body weight, dissolved in sterile Ringer’s solution) were injected once intraperitoneal (i.p.), using identical lot numbers and stock solutions. The vehicle control groups were injected with equal volume of sterile Ringer’s solution. The whole pancreas and part of the liver were resected as described previously^5,6,31^. Mouse blood samples were collected by cardiac puncture. Serum amylase and lipase were determined by the institutional blood analysis center at the Heidelberg University Hospital.

### Genotyping

DNA extraction was performed according to the protocol of the Fast Tissue-to-PCR Kit, using mouse ears. Two μl DNA extracts were added to a 20 μl PCR reaction system containing Dream Taq PCR Master Mix primers and nuclease-free water (Thermo Fisher Scientific, Waltham, MA, USA). DNA amplification was conducted in a Mastercycler personal PCR machine (Eppendorf, Germany), followed by separation on a 2% agarose gel containing DNA Stain G (SERVA, Heidelberg, Germany) under standard DNA electrophoresis conditions and UV illuminator visibility. The primers used in the PCR reaction are listed: Atg7 forward primer: 5′-TGGCTGCT-ACTTCTGCAATGATGT-3′, reverse primer: 5′-CAG-GACAGAGACCATCAGCTCCAC-3′; p48-cre forward primer: 5′-ACCGTCAGTACGTGAGATATCTT-3′, reverse primer: 5′-ACCTGAAGATGTTCGCGATTATCT-3′. We did not show genotyping from the Lamp2 mice.

### Immunofluorescence

IF was conducted using FFPE pancreatic tissue sections and performed as described in detail previously^5,6,30,31,37^. All IF images were acquired using the TissueFAXS system and further analyzed by the StrataQuest software (TissueGnostics, Vienna, Austria), allowing the quantification of target-protein positive cell numbers from DAPI positive cells or amylase-specific positive cells (indicating pancreatic acinar cells), as previously described in detail^5,6,31^. Infiltrated MPO-positive monocytes/ neutrophils were determined by the numbers of positive cell per mm^2^ tissue. All other chemicals were from Sigma-Aldrich (St. Louis, USA) or ROTH (Karlsruhe, Germany), if not stated otherwise.

### Immunohistochemistry

LPS staining for bacteria were conducted using the automated slide stainer BOND RXm (Leica) using the Bond polymer refine detection kit, according to manufacturer’s instructions and as described recently^38^.

FFPE pancreatic tissue was cut into 4-μm-thick sections and subject for heat induced epitope retrieval (HIER) at pH 6 for 20 min heating step with the epitope retrieval solution 1 (BOND). Gram negative were stained with anti-LPS Core (1:1000 dilution), following the counterstaining. Images were scanned and analyzed using the StrataQuest software (TissueGnostics), allowing the quantitation of the total cell numbers and DAB-positive cells. In FACS-like scattergrams, the cells were plotted according to their DAB positivity intensity versus their hematoxylin counterstain positive cells in the entire tissue.

### Endotoxin quantitation

Endotoxin or LPS levels in tissue homogenates samples were determined using the Chromogenic Endotoxin Quant Kit (Thermo Fisher Scientific) in microplate following the manufacturer’s instructions. Briefly, 100 µg total tissue protein extract, standards and blanks were incubated with the amebocyte lysate reagent and chromogenic substrate (SUB). After adding of 25% acetic acid to stop the reaction, the absorbance was determined at 405 nm (Microplate MultiKan FC, Thermo Scientific).

### Statistical analysis

*P* values were calculated using the two-sample unpaired/independent samples t-tests with Welch’s correction. The analysis of possible outliers was performed for all column data sets. Results were considered to significant when *p* value ≤ 0.05, indicated with *, and were presented as mean ± SEM (standard error of the mean) or standardization to controls as indicated with the significance score (*< 0.05; **< 0.01; ***< 0.001; ****< 0.0001) in the figure legends. GraphPad Prism 6 software was applied for the statistical calculations.

## Results

### Local deletion of *Atg7* impairs autophagy in the pancreas

**Fig. 1:**
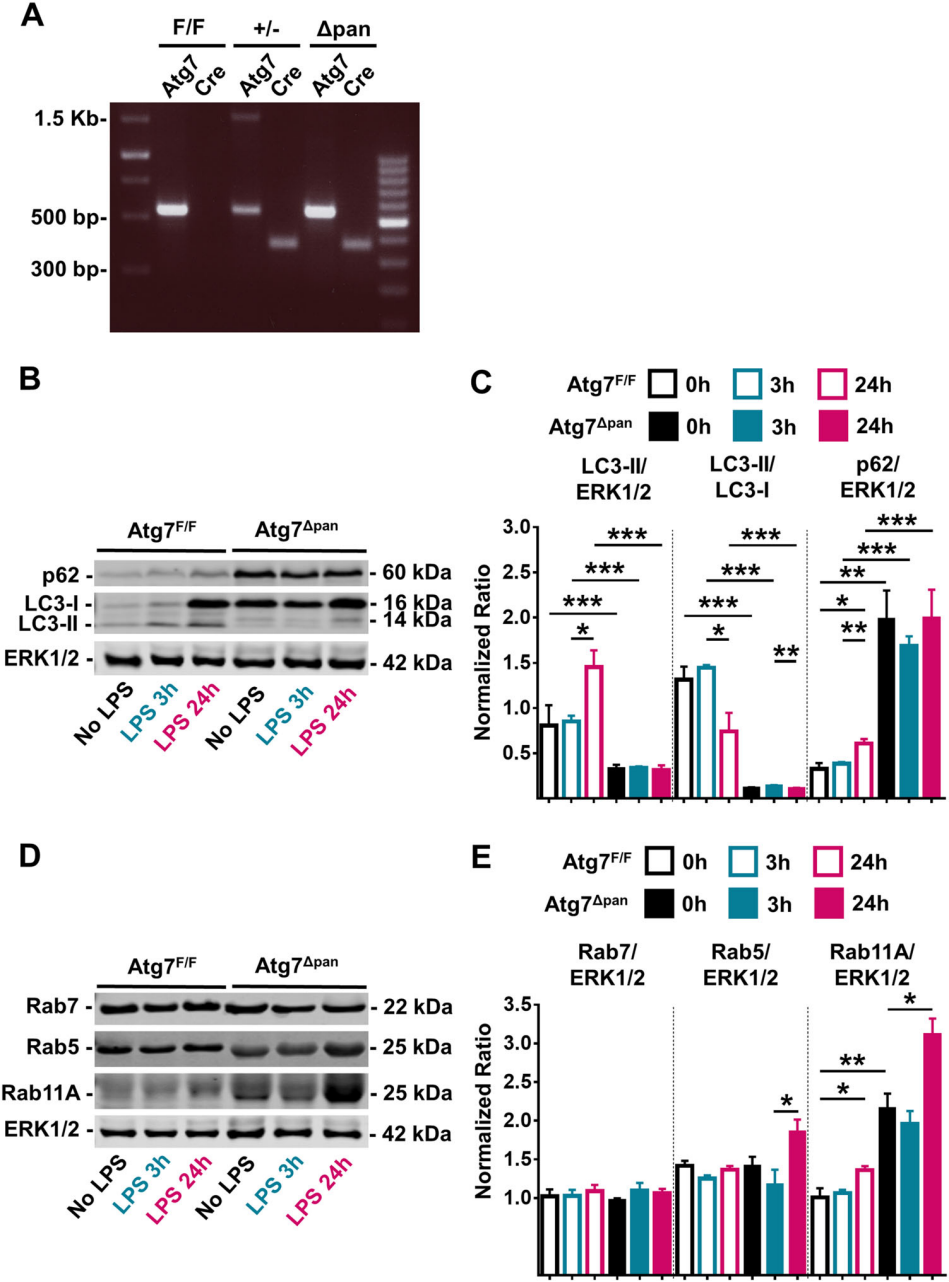
Assesment of pancreatic autophagy and endosomal function after LPS injection. **a** Representative PCR agarose gel electrophorese image of PCR products obtained from the DNA of mice with the indicated genotypes. PCR fragments for Atg7^F/F^ mice: 500bp (Atg7), blank (Cre); Atg7^+/−^ mice: 1.5 Kb and 500bp (Atg7), 350bp (Cre); Atg7^Δpan^ mice: 500bp (Atg7), 350bp (Cre). Evaluation of autophagic activity after LPS injection. **b** Representative autophagy immunoblots detecting LC3, p62, and ERK1/2 (loading control) in the pancreas of Atg7^F/F^ and Atg7^Δpan^ mice. **c** The normalized values of LC3-II, LC3-II/LC3-I, and p62 were quantified as means ± SEM of 4 animals per group. **d** Increased recycling endosome Rab11A after LPS. Representative immunoblots of Rab7, Rab5, Rab11A and ERK1/2 (loading control) in the pancreas of Atg7^F/F^ and Atg7^Δpan^ mice. **e** The normalized values of Rab7, Rab5, and Rab11A were quantified as means ± SEM of 4 animals per group, *p < 0.05, **p < 0.01, ***p < 0.001, ****p < 0.0001.

Floxed *Atg7* was specifically deleted by a *Cre* recombinase constitutively expressed in pancreatic epithelial cells under the control of the p48 promoter (Fig. 1a). ATG7 protein was present in the livers of both Atg7^Δpan^ and Atg7^F/F^ mice, and in the pancreas of Atg7^F/F^ mice, but almost undetectable in the pancreas of Atg7^Δpan^ mice by WB (Supplementary Fig. 1A). The deletion of *Atg7* was accompanied by reduced expression of ATG5, significantly reduced conversion of LC3-I to LC3-II, as well as marked accumulation of the autophagic substrate sequestosome-1 (SQTM1, best known as p62) in the pancreata of Atg7^Δpan^ mice. No such changes were found in the liver of Atg7^Δpan^ mice, confirming organ specificity of the knockout (Supplementary Fig. 1A).

### Activation of autophagy in the pancreas of Atg7^F/F^ mice after LPS

To investigate whether endotoxemia affected pancreatic autophagy, we injected LPS systemically via the intraperitoneal (i.p.) route into mice and then determined the levels of LC3-II and p62 in the pancreas. We detected an increase in LC3-II expression in Atg7 knockout mice 24 h after LPS administration but not at basal time or 3 h after LPS administration. The basal expression level of LC3-I was significantly higher in the Atg7 mice, which is similar to that in control mice 24 h after LPS administration. The expression of LC3-I and LC3-II was increased in control mice 24 h after LPS administration. In the Atg7 mice, the LC3-II fragment was more abundant as compared to controls or 3 h after LPS administration. Whilst the ratio with ERK1/2 did not show any significant difference, the ratio with LC3-I was significantly reduced and thus LC3-II was increased 24 h after LPS administration in the Atg7 mice. Our results suggest that autophagy inhibition or induction by the modulation of LC3 lipidation may be facilitated by an Atg7-dependent or independent pathway. In Atg7^Δpan^ mice, LPS administration did not activate autophagy (Fig. 1b, c) but stimulated the expression levels of the early endosomal marker Rab5 and the recycling endosomal marker Rab11a. The late endosomal marker Rab7 expression was not altered by LPS administration (Fig. 1d, e). In autophagy-competent Atg7^F/F^ control mice, the normalized value of pancreatic LC3-II increased 24 h after LPS in Atg7^F/F^ mice (*p* = 0.0664), suggesting an increase in autophagosome formation 24 h after LPS (Fig. 1c). However, the normalized ratio of LC3-II/LC3-I decreased (*p* = 0.0626) 24 h after LPS and simultaneously, the normalized value of p62 significantly increased (*p* = 0.0137) 24 h after LPS injection (Fig. 1c). Rab11b expression was also increased 24 h after LPS injection, while the expression of Rab7 and Rab5 was not influenced by LPS administration (Fig. 1e**)**. Loss of Atg7 seem to accumulate recycle endosomes after LPS. These results suggest that autophagy is initiated but not executed after LPS in Atg7^F/F^ mice, in line with a prior report^6^.

### Atg7^Δpan^ mice exhibit features of CP and increased susceptibility to LPS-induced pancreatic damage

**Fig. 2:**
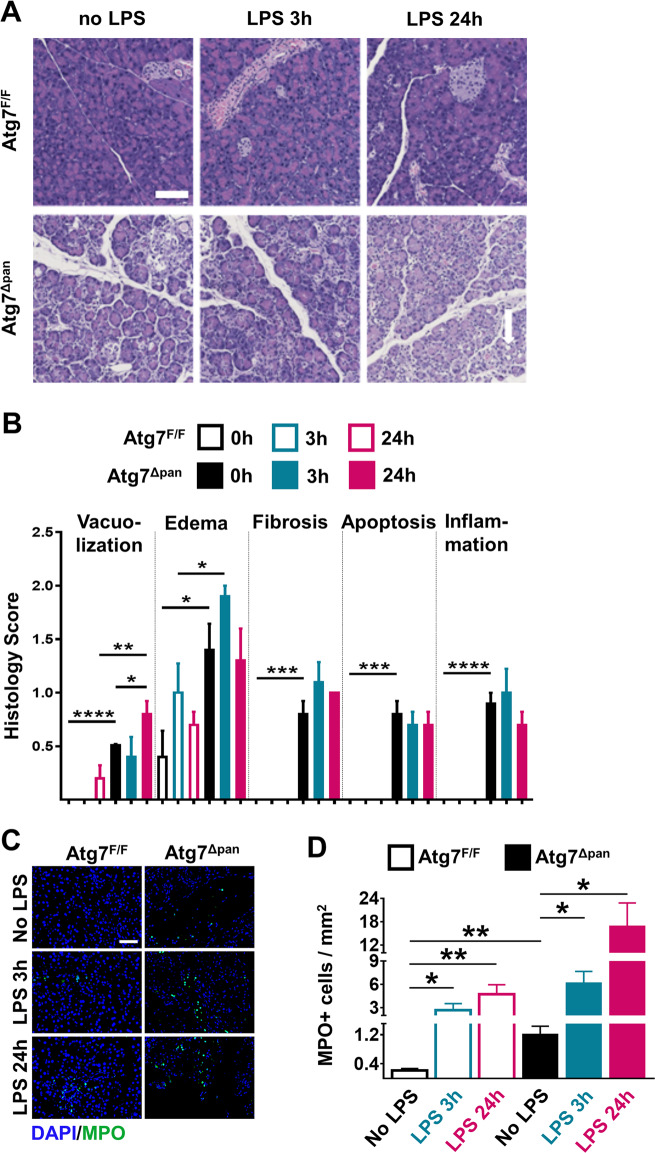
Histopathological evaluation of the mouse pancreatic tissue. **a** Representative H&E staining of pancreatic tissue images from Atg7^F/F^ and Atg7^Δpan^ mice tissue sections with and without LPS (×20 objective; scale bar = 50 μm). **b** Histopathological quantitation of the indicated features of pancreatic damage and inflammation, 3 and 24 h after LPS injection in Atg7^F/F^ and Atg7^Δpan^ mice. The scores were plotted as means ± SEM for each group (*n* = 5). **c** Representative MPO IF images (×20 objective; Scale bar = 50 µm) stained for DAPI (blue) and MPO (green). **d** The density of MPO-positive cells infiltrating the pancreas was quantified as means ± SEM, *n* = 4–5 mice for each group. **d** *p < 0.05, **p < 0.01, ***p < 0.001, ****p < 0.0001.

**Fig. 3:**
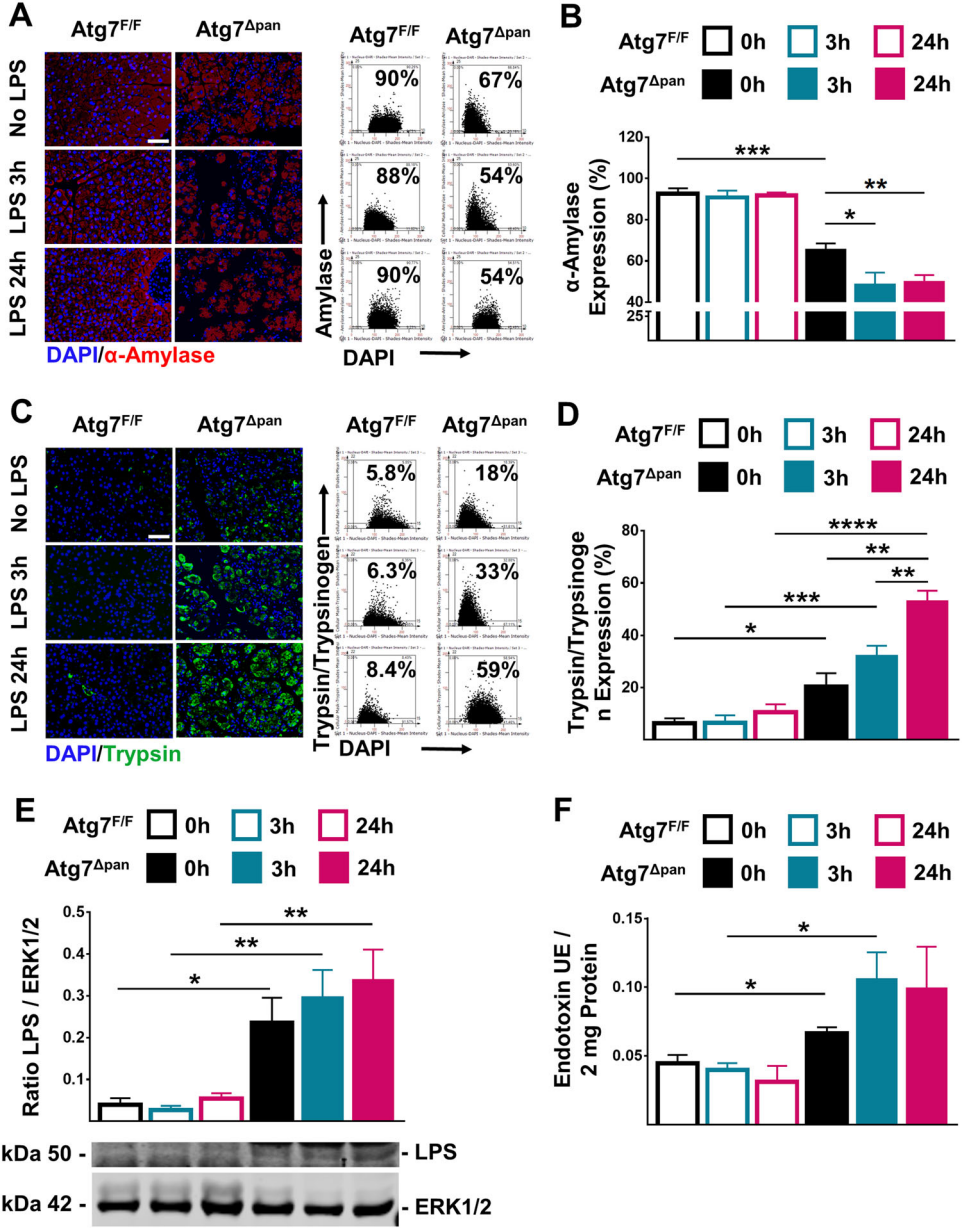
Evaluation of pancreatic function. **a** Representative IF images of pancreatic tissue stained for α-amylase (red). **b** Pancreatic α-amylase expression was determined by FACS-like immunofluorescence (IF) quantitation and values were plotted as means ± SEM for each group (*n* = 5–6). **c** Representative IF images of pancreatic tissue stained for Trypsinogen/Trypsin (green) and DAPI (blue). **d** Trypsinogen/Trypsin expression was quantitated as means ± SEM for each group (*n* = 4–5). **e** Evaluation of pancreatic tissue LPS levels by immunoblot. LPS levels were determined as the ratio of LPS over ERK1/2 (means ± SEM of four animals per group) in Atg7^F/F^ and Atg7^Δpan^ mice. **f** Quantitation of LPS levels in pancreatic tissue homogenate of Atg7^F/F^ and Atg7^Δpan^ mice using a commercial assay. Scale bars = 50 µm. *p < 0.05; **p < 0.01; ***p < 0.001, ****p < 0.0001.

As compared to autophagy-competent Atg7^F/F^ control mice, 12-week-old *Atg7*^Δpan^ mice exhibited a reduced body weight (Supplementary Fig. 1B), consistent with a prior report^5^. Normal mice are able to tolerate doses well of up to 25 mg/kg LPS while Atg7^Δpan^ mice barely tolerated 5 mg/kg)^39^. At this dose, Atg7^F/F^ mice showed no abnormal behavior except minor diarrhea while, Atg7^Δpan^ mice manifested severe body weight loss 24 h after LPS (Supplementary Fig. 1C). Histopathological evaluation of hematoxylin-eosin stained pancreatic tissues revealed multiple signs of inflammation in autophagy-deficient *Atg7*^Δpan^ as compared to autophagy-sufficient *Atg7*^F/F^ control samples at baseline: vacuolization of acinar cells, tissue edema, fibrosis, apoptosis, and infiltration by inflammatory cells. These signs of inflammation were barely influenced by LPS injection in autophagy-deficient *Atg7*^Δpan^ pancreata. In contrast, LPS injection induced a significant vacuolization of the autophagy-sufficient *Atg7*^F/F^ pancreas within 24 h (Fig. 2a, b), in line with the interpretation that autophagy initiation has occurred in response to LPS **(**Fig. 1b, c**)**. Myeloperoxidase (MPO), an early marker for infiltration by neutrophil granulocytes and monocytes, significantly increased 3 h after LPS injection and showed an additional elevation 24 h later in both *Atg7*^F/F^ and *Atg7*^Δpan^ mice. However, *Atg7*^Δpan^ mice exhibited higher infiltration by MP0^+^ cells than *Atg7*^F/F^ controls at baseline (Fig. 2c, d). Of note, *Atg7*^Δpan^ pancreata contained less α-amylase than Atg7^F/F^ mice, indicating loss of pancreatic function that was further exacerbated by LPS injection, as determined by quantitative immunofluorescence (Fig. 3a, b) or by immunoblot (Supplementary Fig. 1D). While serum α-amylase similarly increased in LPS-injected *Atg7*^F/F^ and *Atg7*^Δpan^ mice, serum lipase increased in *Atg7*^Δpan^ but not in *Atg7*^F/F^ mice (Supplementary Fig. 2A, B). Serum glucose decreased while serum LDH increased after LPS-injected *Atg7*^F/F^ and *Atg7*^Δpan^ mice although the basal serum glucose level is increased *Atg7*^Δpan^ mice (Supplementary Fig. 2C, D).

Trypsin/trypsinogen expression showed no significant change 3 or 24 h after LPS injection in *Atg7*^F/F^ control mice. Autophagy-deficient *Atg7*^Δpan^ mice exhibited an increased expression level of trypsin/trypsinogen at baseline, and this phenomenon was amplified by exposure to LPS (Fig. 3c, d), again suggesting that autophagy-deficient *Atg7*^Δpan^ mice undergo more severe pancreatic injury after LPS.

### LPS is increased in experimental pancreatic tissue samples

We found that bacterial LPS was increased in *Atg7*^Δpan^ pancreata compared to *Atg7*^F/F^ controls even before LPS injection, as determined by two alternative techniques, namely immunoblotting and biochemical endotoxin measurements. LPS injection failed to increase local LPS levels further **(**Fig. 3e, f**)**.

### LPS is present in human CP tissues

**Fig. 4:**
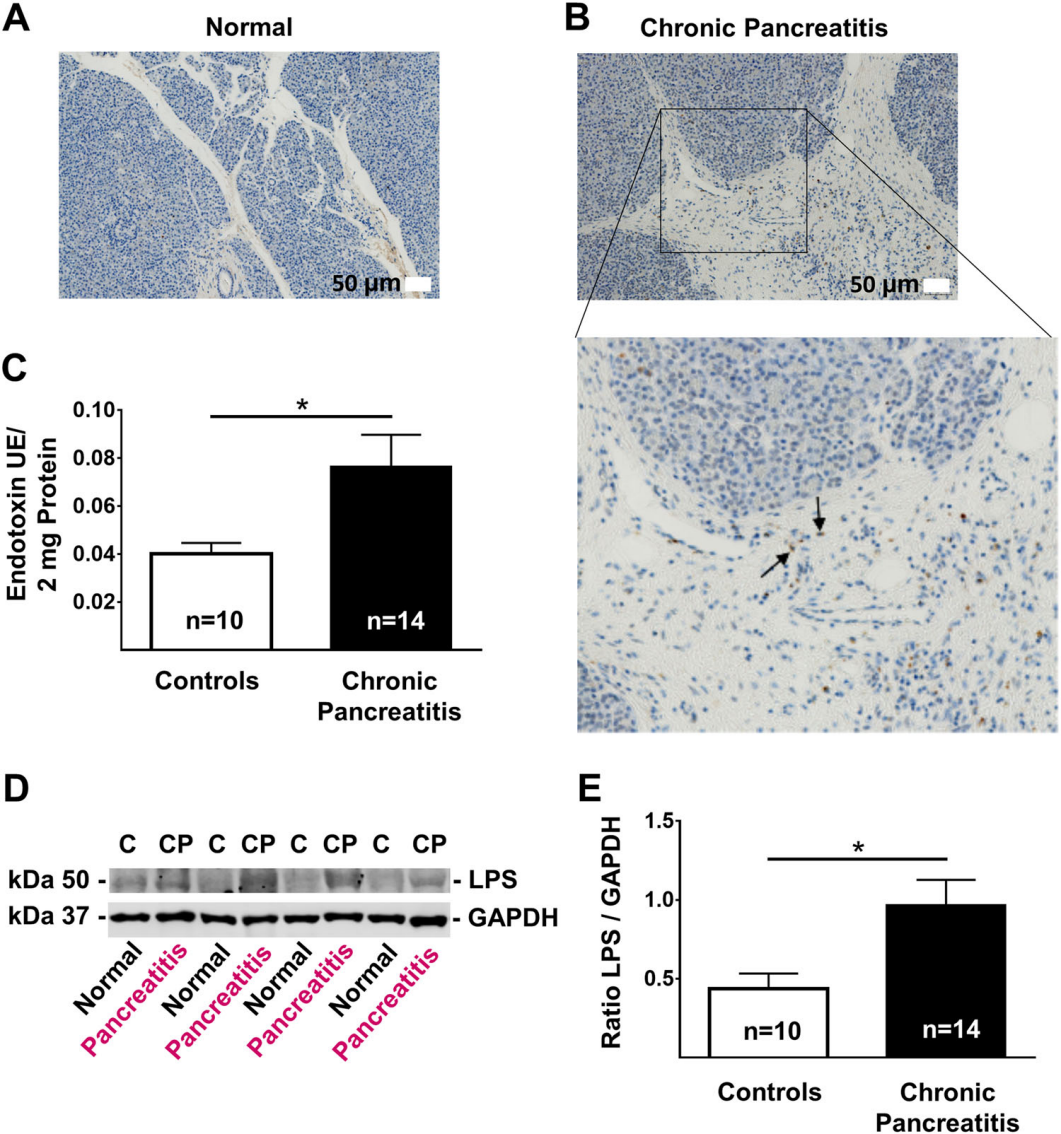
Endotoxin level in human chronic pancreatitis. **a** Representative IHC stained for LPS in pancreatic tissue form a normal donor. **b** Representative IHC stained for LPS in pancreatic tissue form a chronic pancreatitis patient. Black arrows indicate LPS-positive stained cell within the pancreatic tissue. **c** Quantitation of LPS levels in human normal and pancreatitis tissue homogenate using a commercial kit (means ± SEM). **d** Representative immunoblot images stained for LPS and GAPDH (loading control) in normal and chronic pancreatitis tissue. **e** The intensity of bands was quantified at the ratio of LPS over GAPDH means ± SEM). Scale bar = 50 µm. *p < 0.05.

Histopathology severity score in CP and healthy donor patients were summarized in Table 1. IHC detected LPS-positive structures in 14 human pancreatic tissues but were not detectable in 10 donor control pancreata **(**Fig. 4a, b**)**. By using chromogenic endotoxin quantitation kit LPS significantly increase by 1.9-fold (*p* = 0.0328) in CP compared to controls **(**Fig. 4c**)**. WB analysis also showed a significantly increase of LPS by 2.2-fold (*p* = 0.0177) in CP compared to controls **(**Fig. 4d, e**)**. Hence, both pancreatitis induced by genetic autophagy deficiency in mice and naturally occurring pancreatitis in humans is associated with an increase in LPS levels within the inflamed tissue.

### LPS in pancreatic tissue samples is coupled to increased TLR4 activation

**Fig. 5:**
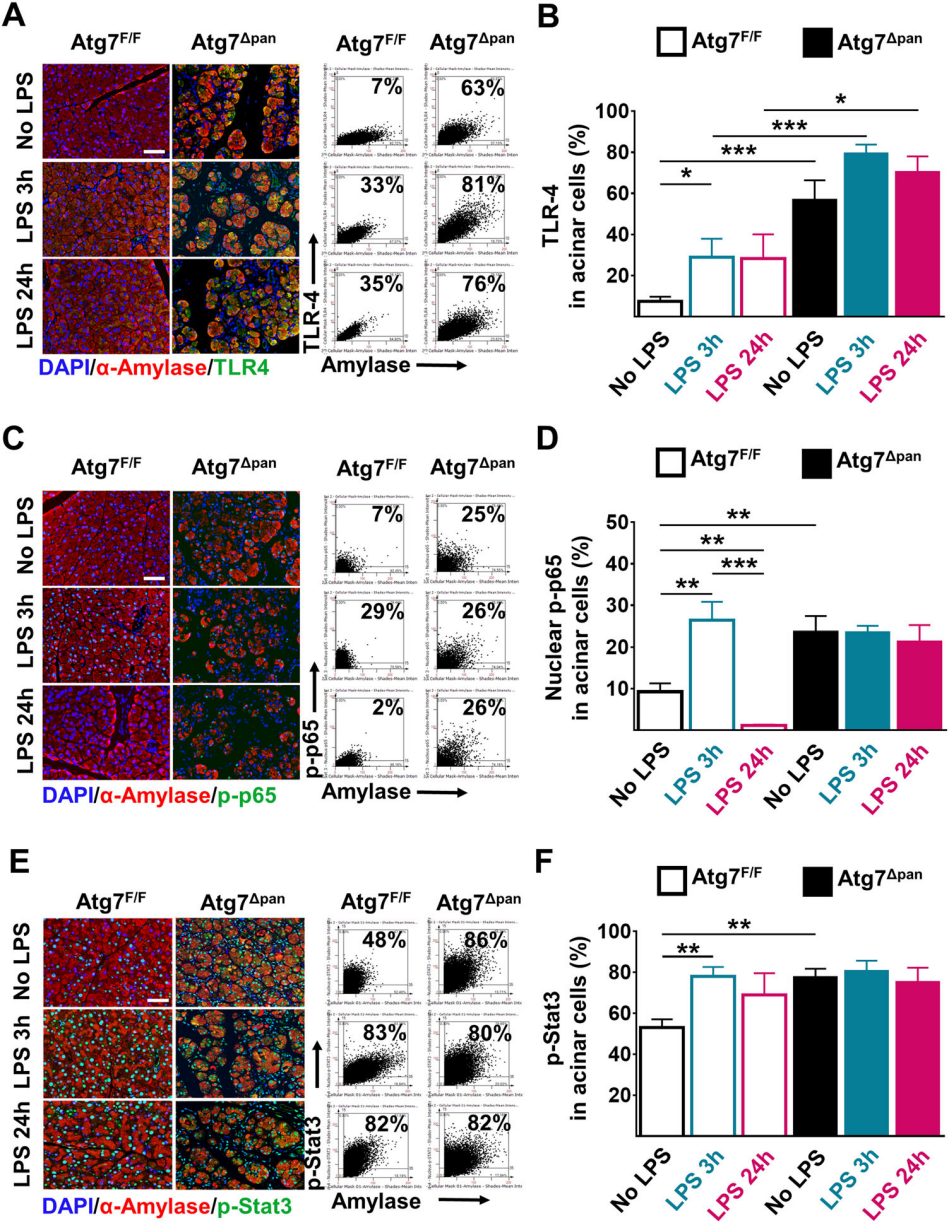
Evaluation of pancreatic LPS signalling. **a** Representative IF colocalization images stained for TLR4 (green), α-amylase (red) and DAPI (blue), as well as representative FACS-like scattergrams of co-expression quantitation. **b** Pancreatic expression of TLR4 in acinar cells from Atg7^F/F^ and Atg7^Δpan^ mice, 3 and 24h after LPS were determined by IF FACS-like quantitation and plotted as means ± SEM (*n* = 5 per group). **c** Representative IF colocalization images stained for phosho-p65 (green), α-amylase (red) and DAPI (blue), as well as representative FACS-like scattergrams. **d** Pancreatic expression of nuclear phosho-p65 in acinar cells from Atg7^F/F^ and Atg7^Δpan^ mice, 3 and 24h after LPS were plotted as means ± SEM (*n* = 4–5). **e** Representative IF colocalization images stained for p-Stat-3 (green), α-amylase (red) and DAPI (blue) with FACS-like scattergrams. **f** Pancreatic expression of p-Stat-3 determined by IF FACS-like quantitation and plotted as means ± SEM (*n* = 4–5). Scale bar = 50 µm. *p < 0.05; **p < 0.01; ***p < 0.001.

The expression of acinar cell TLR4 in *Atg7*^F/F^ mice was significantly increased 3 h after LPS administration (*p* = 0.048) but did not reach significance at 24 h post LPS administration (Fig. 5a). In *Atg7*^Δpan^ mice, acinar cell TLR4 expression was constitutively elevated as compared to *Atg7*^F/F^ controls, with no significant further induction by LPS (Fig. 5b). Upon LPS binding, TLR4 signaling results in the activation of the NF-κB pathway^40^ as well as the activating phosphorylation of STAT3^41,42^. Accordingly, LPS injection caused a transient (3 h) increase in the phosphorylation and nuclear translocation of p65 in *Atg7*^F/F^ controls, while the activation of the NF-κB pathway appeared to be constitutive in *Atg7*^Δpan^ mice (Fig. 5c, d). Similarly, phosphorylation of STAT3 could be stimulated by LPS in *Atg7*^F/F^ control pancreata, but was constitutively activated in *Atg7*^Δpan^ mice and no further induced by LPS in this latter genotype (Fig. 5e, f). Altogether, these results suggest that LPS-induced pancreatic injury leads to the activation of TLR4 signaling pathway with its downstream effectors NF-κB and STAT3.

### Acinar cell apoptosis and necroptosis in *Atg7*^F/F^ and *Atg7*^Δpan^ mice

**Fig. 6:**
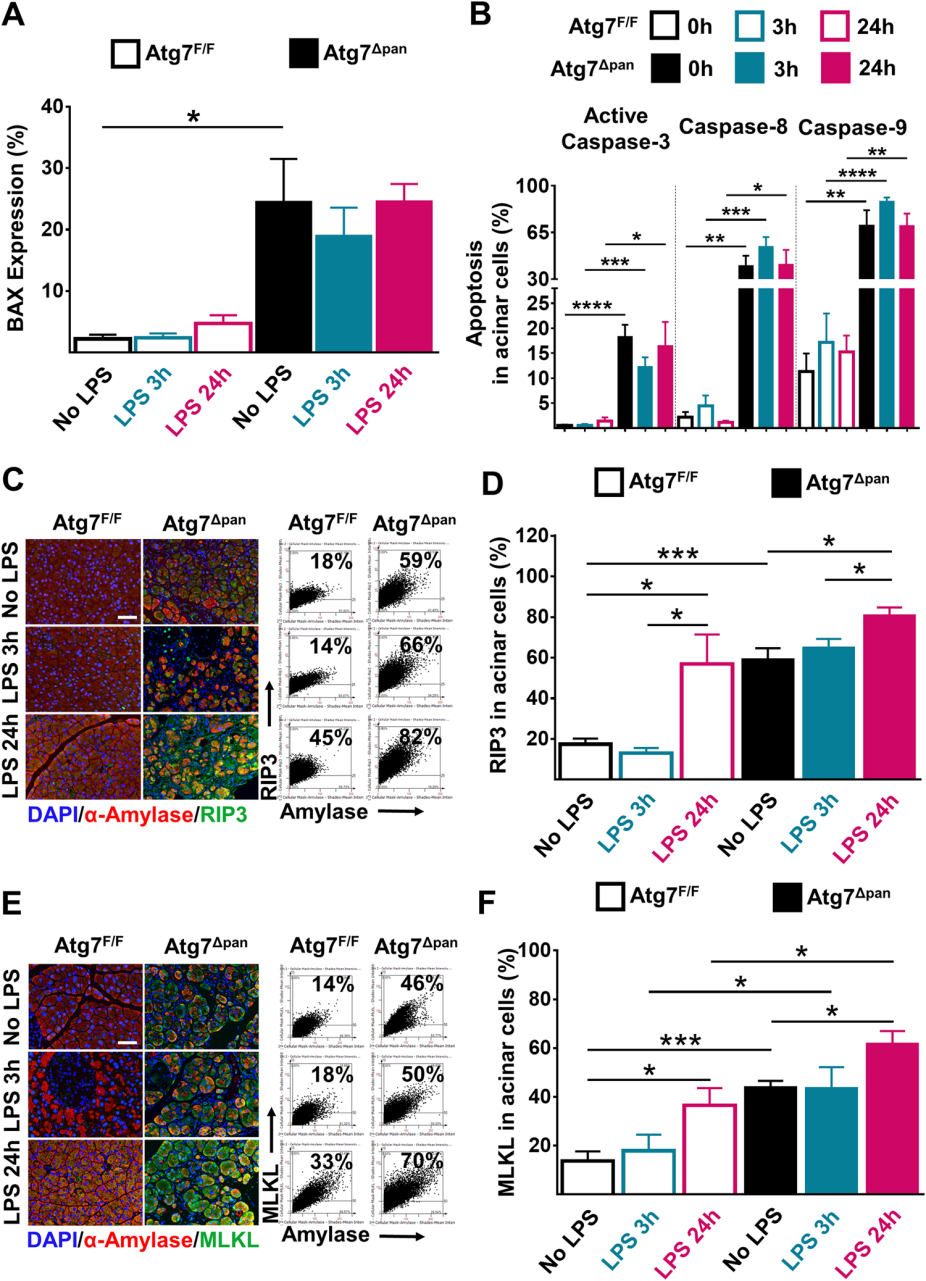
Estimation of pancreatic cell death after LPS exposure. **a** Pancreatic BAX expression was determined by FACS-like IF quantitation and plotted as means ± SEM (*n* = 5). **b** Pancreatic expression levels of active Caspase-3, Caspase-8, and Caspase-9 in acinar cells were determined by FACS-like IF quantification and plotted as means ± SEM (*n* = 4–5). **c** Representative IF colocalization images stained for RIP3 (green), α-Amylase (red), and DAPI (blue), as well as representative FACS-like scattergrams quantification of colocalization of RIP3 and α-amylase. **d** Pancreatic expression levels of RIP3 in acinar cells were determined by IF FACS-like quantitation and plotted as means ± SEM (*n* = 4–5). **e** Representative IF colocalization images stained for MLKL (green), α-amylase (red), and DAPI (blue), as well as representative FACS-like scattergrams quantification. **f** Expression levels of MLKL in acinar cells of the pancreas determined by IF FACS-like quantitation, plotted as means ± SEM (*n* = 4–5 per group). Scale bar = 50µm, *p < 0.05, **p < 0.01, ***p < 0.001, ****p < 0.0001.

The pancreatic expression of apoptotic Bax, active caspase-3, caspase-8, and caspase-9 were elevated in *Atg*7^Δpan^ mice as compared to *Atg7*^F/F^ controls, as determined by quantitative immunofluorescence microscopy, focusing on pancreatic acinar (α-amylase-positive) cells. Interestingly, LPS failed to induce these apoptosis markers in both *Atg7*^F/F^ and *Atg7*^Δpan^ mice (Fig. 6a, b Supplementary Fig. 3A–D**)**. The expression of necroptosis-related molecules RIP3 and MLKL in pancreatic acinar cells was significantly augmented in Atg7^Δpan^ mice compared to Atg7^F/F^. LPS injection enhanced this sign of necroptosis further, both in *Atg7*^F/F^ and *Atg7*^Δpan^ mice, as determined by quantitative immunofluorescence microscopy (Fig. 6c–f) and confirmed by immunoblot (Supplementary Fig. 4A, B). These results suggest an increase in constitutive apoptotic and necrotic cell death in autophagy-deficient pancreata. Exogenous LPS can stimulate the necroptotic but the apoptotic destruction of the parenchyma of the exocrine pancreas.

### LPS-stimulated inflammation in the pancreas from *Atg7*^Δpan^ mice

**Fig. 7:**
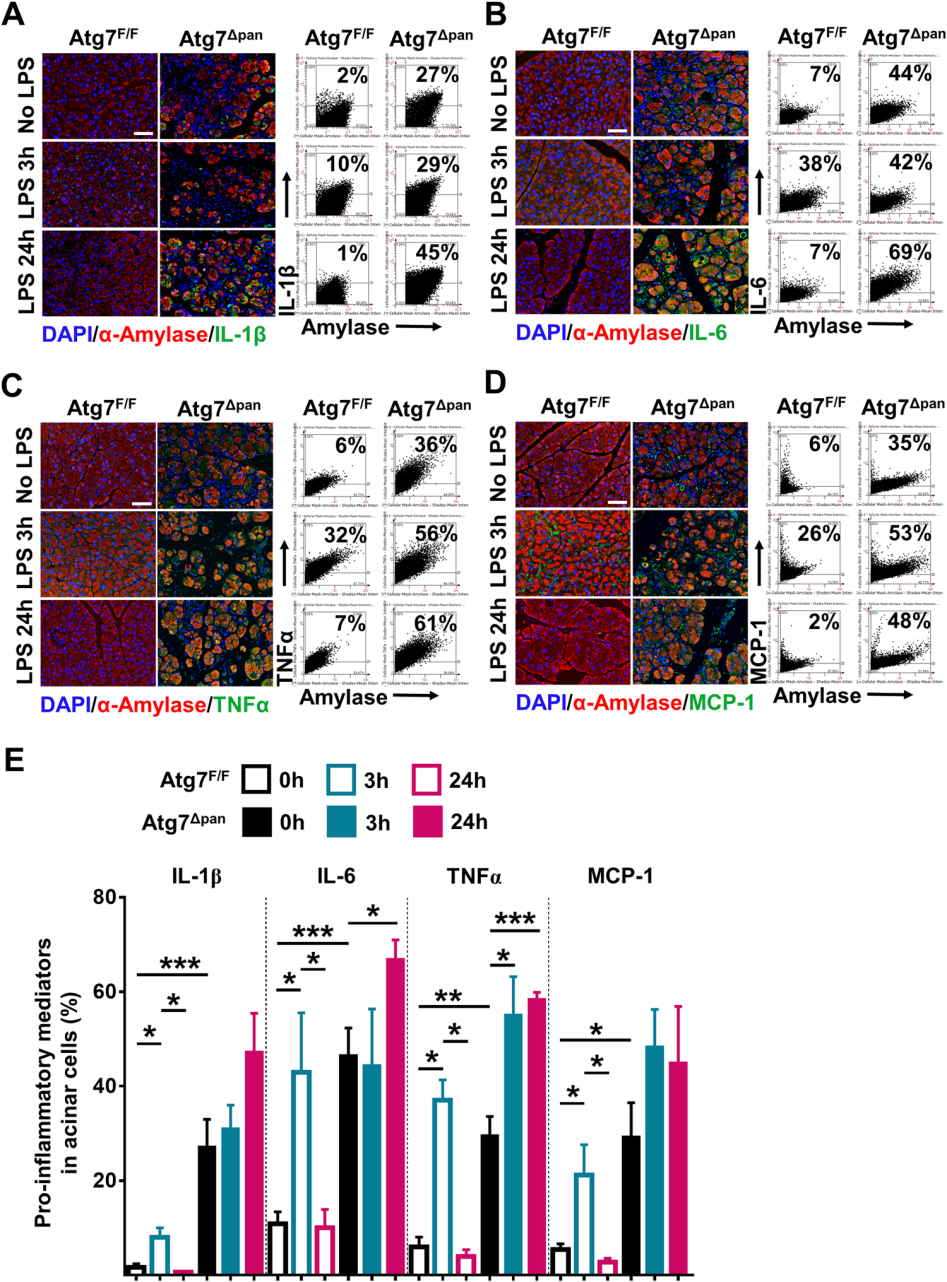
Enhanced pro-inflammatory mediators in acinar cells after LPS injection into Atg7^Δpan^ mice. **a** Representative IF colocalization images stained for IL-1β (green), α-Amylase (red), and DAPI (blue), as well as representative FACS-like scattergrams quantification of colocalization of IL-1β and α-Amylase. **b** Representative IF colocalization images stained for IL-6 (green), α-amylase (red), and DAPI (blue), as well as quantification of colocalization of IL-6 and α-amylase. **c** Representative IF colocalization images stained for TNFα (green), α-amylase (red), and DAPI (blue), as well as representative FACS-like scattergrams quantification of the colocalization of TNFα and α-amylase. **d** Representative IF colocalization images stained for MCP-1 (green), α-amylase (red), and DAPI (blue), and quantification of colocalization of MCP-1 and α-Amylase. **e** Pancreatic expression levels of pro-inflammatory mediators (IL-1β, IL-6, TNFα, and MCP-1) in acinar cells from Atg7^F/F^ controls and Atg7^Δpan^ mice 3 and 24 h after LPS were determined by FACS-like IF quantitation and plotted as means ± SEM (*n* = 4–5 per group). Scale bar = 50 µm, *p < 0.05, **p < 0.01, ***p < 0.001.

We investigated the intra-acinar cell expression of inflammatory mediators by using IF colocalization quantitation with α-Amylase. Atg7^Δpan^ mice revealed higher basal expression levels of pro-inflammatory IL-1β **(**Fig. 7a**)**, IL-6, **(**Fig. 7b**)**, TNFα, **(**Fig. 7c**)**, and MCP-1 **(**Fig. 7d**)** compared to Atg7^F/F^ controls. The expression of pro-inflammatory mediators strongly increased in Atg7^F/F^ mice 3 h after LPS and decreased to basal level 24 h later, as shown previously^30,31^. In contrast, in autophagy-deficiency Atg7^Δpan^ mice, all pro-inflammatory mediators remained markedly elevated 24 h after LPS compared to no LPS injection group (Fig. 7e**)**.

The role of anti-inflammatory mediators was further investigated. Atg7^Δpan^ mice revealed higher basal expression levels of anti-inflammatory IL-10 and TGF-β compared to Atg7^F/F^ controls **(**Supplementary Fig. 4C, D**)**. The expression of anti-inflammatory mediators strongly increased 3 h after LPS and remained higher 24 h later in Atg7^F/F^ mice, in contrast to previous research^30,31^. In autophagy-deficient mice, all anti-inflammatory mediators remained high but showed no alteration after LPS (Supplementary Fig. 4E**)**. Our findings suggested that Atg7^Δpan^ mice lose the ability to maintain the balance between pro- and anti-inflammatory mediators to control inflammation, commensurate with the higher suscep-tibility of autophagy-deficient mice to LPS-induced pancreatitis.

Furthermore, the expression level of pancreatic p-JNK was higher in Atg7^Δpan^ mice compared to Atg7^F/F^ controls and was significantly increased 24 h after LPS in both mouse strains (Supplementary Fig. 5A, B). Interestingly, p-ERK expression increased 3 h after LPS and decreased 24 h later in the Atg7^F/F^ control mice, while p-ERK expression only showed a similar tendency without reaching significance in Atg7^Δpan^ mice after LPS. The basal expression level of pancreatic p-c-Jun significantly increased in Atg7^Δpan^ mice compared to the Atg7^F/F^ mice, and its expression significantly increased 3 h and decreased 24 h after LPS in both Atg7^F/F^ and Atg7^Δpan^ mice (Supplementary Fig. 5A, B).

### Loss of global LAMP2 exacerbates LPS-induced pancreatic injury

Consistent with the *Atg7*^Δpan^ mice, Lamp2^F/F^ control mice are able to tolerate doses of up to 25 mg/kg LPS, while Lamp2^y/−^ barely survive 1.5 mg/kg LPS at an age of 25 weeks^39^. As compared to age-matched autophagy-competent LAMP2 control mice, 25-week-old *Lamp2*^y/−^ mice exhibited also strong vacuolization of acinar cells and tissue edema (Supplementary Fig. 6A, B), reduced body weight (Supplementary Fig. 6D) and increased serum signs of pancreatic damage after LPS (Supplementary Fig. 6C–E). LPS injection increased also acinar cell TLR4 and p65 expression, while α-amylase was only marginal reduced (Supplementary Fig. 7A–C). Similar to Atg7^Δpan^ mice, loss of LAMP2 increased necroptosis, which is also exacerbated after LPS injection (Supplementary Fig. 8A–D). The inflammatory response in acinar cell showed also that loss of LAMP2 accelerated inflammatory markers after LPS. LPS (Supplementary Fig. 9A–C). These results confirm that autophagy deficiency, caused by local Atg7 deletion (*Atg7*^Δpan^) or global Lamp2 deletion (*Lamp2*^y/−^) exacerbates LPS-induced pancreatic injury.

## Discussion

Previous work have confirmed that pancreatic acinar cells respond to LPS during the progression of mild, sub-clinical acute pancreatitis^30^. Moreover, there is increasing evidence for metabolic endotoxemia contributing to the pathogenesis of CP due to gut microbial dysbiosis^22–24,29^. CP is also associated with reduced levels of Atg7, Atg5, LC3, and Lamp-2 as well as increased p62, indicating reduced autophagy in CP tissue^4–6^. The present research shows for the first time the presence of LPS in human CP tissue. Furthermore it elucidates the cytoprotective and anti-inflammatory role of autophagy in acinar cells responding to LPS, using a mouse model in which the essential autophagy protein ATG7 is selectively removed in the pancreas. Histopathological examination, evaluation of weight loss, measurements of serum parameters, and quantitation of α-amylase and trypsin/trypsinogen in the pancreatic tissue demonstrated that LPS-induced endotoxemia leads to pancreatic injury in autophagy-deficient Atg7^Δpan^ mice but not in controls, revealing the protective role of autophagy/xenophagy in acinar cells exposed to endotoxin.

Normal mice can tolerate i.p. injections of up to 25–30 mg/kg LPS^39^, depending on the LPS batch, while only 5 mg/kg LPS had severe toxic effects on pancreatic autophagy-deficient mice. Similar results were obtained for LAMP-2 deficient mice (that are deficient in their autophagic and lysosomal activity in all tissues), which are also highly susceptible to LPS-induced pancreatitis. Thus, the loss of pancreatic autophagy subsequent to the local or global knockout of Atg7 or Lamp2, respectively, increases the susceptibility to develop pancreatic injury after LPS administration. Twelve-week-old Atg7^Δpan^ mice showed increased activation of trypsin, RIP3, and MLKL at baseline, but more so in response to LPS, suggesting that the depletion of Atg7 from acinar cells is sufficient to predispose the tissue to pancreatitis.

Although pancreatic apoptosis was over-activated se-condary to the suppression of autophagy, LPS exposure was unable to further enhance apoptosis. Acinar cell apoptosis triggered by LPS has been reported to be associated with acute pancreatic injury in clinical and experimental models^6,43,44^. However, acinar cell apoptosis has also been suggested to be a beneficial response to acinar injury during pancreatitis, as opposed to acinar cell necrosis^45,46^. As a form of regulated necrosis, acinar cell necroptosis was activated 24 h after LPS in both normal mice and Atg7^Δpan^ mice. One recent study supports the concept that LPS induces RIP3 and MLKL-dependent necroptosis in macrophages^47^. Another study suggested that RIP3 activity may actually suppress apoptosis^48^, providing a possible explanation why LPS stimulates necroptosis with RIP3 (and MLKL) activation without exacerbating acinar cell apoptosis. Irrespective of these mechanistic considerations, it appears that autophagy-inhibited necroptosis/necrosis is more important for mediating excessive death of pancreatic acinar cells during endotoxemia than is apoptosis.

In response to LPS, the TLR4 signaling pathway activates the transcription factors NF-κB and AP-1, thereby enhancing the production of inflammatory cytokines. In ATG7-deficient pancreata, basal TLR4 expression was elevated compared to Atg7^F/F^ controls. Both NF-κB and AP-1 activation mediated by TLR4 pathway were detected in normal mice after LPS administration, while Atg7^Δpan^ mice responded to LPS only by AP-1 activation, presumably through and ERK and JNK-dependent pathway operating downstream of TLR4. However, the exact contribution of NF-κB and AP-1 to LPS-induced, autophagy-repressed pancreatitis requires further investigation.

Our data demonstrate that the exocrine pancreas undergoes local pro- and anti-inflammatory responses after LPS exposure. In Atg7^F/F^ mice, the early pro-inflammatory response in acini seems to be progressively suppressed by a compensatory anti-inflammatory response. Another study reported a similar effect of pro-inflammatory interferon-β and anti-inflammatory cytokine IL-10 in macrophages after LPS treatment in vitro^49^. Considering that pro-inflammatory cytokines are crucially associated with the pathogenesis of acute pancreatic injury^50,51^, the impaired homeostasis between pro- and anti-inflammatory mediators in acinar cells might predispose LPS-challenged autophagy-deficient Atg7^Δpan^ mice to developing more severe pancreatic injury than normal mice.

Autophagy can be induced by various stimuli, including LPS^10^, in multiple organs such as the lung, intestine, and liver^17,18,52^. In this study, autophagy signaling was activated, yet the completion of autophagy was blocked 24 h after LPS in the normal pancreas. Indeed, pancreatic expression of LAMP2, a lysosomal membrane protein and essential for the fusion of lysosomes with autophagosomes, is significantly reduced by endotoxemia, explaining the blockade of autophagic flux^6^.

The Rab11 family, which is commonly considered as a marker of recycling endosomes, comprises three members, Rab11a, Rab11b, and Rab11c/Rab25. Rab11a, the most well studied of these markers, has been implicated in recycling cell membrane pathways and controls additional processes including phagocytosis^53,54^. Over-expression of wild-type Rab11a inhibits macrophage phagocytosis of apoptotic neutrophils^55^. An increase in Rab11a was detected in this study in autophagy-deficient pancreata and in response to LPS, suggesting that endosomes to be recycled may accumulating and the endosomal pathway cannot further be executed due to the loss of autophagy. Although it is not clear whether this protein contributes to disease pathogenesis, there is an interaction of the endosomal and autophagosomal pathway in the pancreas.

In summary, the present study shows for the first time the presence of LPS in human CP tissue providing supporting evidence for gut microbial dysbiosis leading to metabolic endotoxemia in the pathogenesis of chronic. The present research also provides evidence in favor of the idea that LPS/TLR4-mediated activation of NF-κB and AP-1 is involved in the production of pro- and anti-inflammatory mediators in pancreatic acinar cells. Loss of acinar cell autophagy exacerbated the pro-inflammatory and weakened the anti-inflammatory in responses to endotoxemia in the exocrine pancreas, thereby increasing local tissue damage. It will be valuable to develop strategies to counter the metabolic endotoxemia and pharmacologically stimulate autophagic flux in the pancreas to attenuate the progression of CP.

## Supplementary information

Supplementary Material

Supplementary Fig. 1

Supplementary Fig. 2

Supplementary Fig. 3

Supplementary Fig. 4

Supplementary Fig. 5

Supplementary Fig. 6

Supplementary Fig. 7

Supplementary Fig. 8

Supplementary Fig. 9

## References

[1] Kleeff J (2017). Chronic pancreatitis. Nat. Rev. Dis. Prim..

[2] Whitcomb DC (2016). Chronic pancreatitis: an international draft consensus proposal for a new mechanistic definition. Pancreatology.

[3] Diakopoulos KN (2015). Impaired autophagy induces chronic atrophic pancreatitis in mice via sex- and nutrition-dependent processes. Gastroenterology.

[4] Zhou X (2017). The bile acid receptor FXR attenuates acinar cell autophagy in chronic pancreatitis. Cell Death Discov..

[5] Zhou X (2017). RIP3 attenuates the pancreatic damage induced by deletion of ATG7. Cell Death Dis..

[6] Fortunato F (2009). Impaired autolysosome formation correlates with Lamp-2 depletion: role of apoptosis, autophagy, and necrosis in pancreatitis. Gastroenterology.

[7] Mareninova OA (2009). Impaired autophagic flux mediates acinar cell vacuole formation and trypsinogen activation in rodent models of acute pancreatitis. J. Clin. Invest..

[8] Mareninova OA (2015). Lysosome associated membrane proteins maintain pancreatic acinar cell homeostasis: LAMP-2 deficient mice develop pancreatitis. Cell Mol. Gastroenterol. Hepatol..

[9] Antonucci L (2015). Basal autophagy maintains pancreatic acinar cell homeostasis and protein synthesis and prevents ER stress. Proc. Natl Acad. Sci. USA.

[10] Levine B, Mizushima N, Virgin HW (2011). Autophagy in immunity and inflammation. Nature.

[11] Thorburn A (2018). Autophagy and disease. J. Biol. Chem..

[12] Mizushima N, Yamamoto A, Matsui M, Yoshimori T, Ohsumi Y (2004). In vivo analysis of autophagy in response to nutrient starvation using transgenic mice expressing a fluorescent autophagosome marker. Mol. Biol. Cell.

[13] Fader CM, Sanchez D, Furlan M, Colombo MI (2008). Induction of autophagy promotes fusion of multivesicular bodies with autophagic vacuoles in k562 cells. Traffic.

[14] Gukovskaya AS, Gukovsky I, Algul H, Habtezion A (2017). Autophagy, Inflammation, and Immune Dysfunction in the Pathogenesis of Pancreatitis. Gastroenterology.

[15] Grasso D (2011). Zymophagy, a novel selective autophagy pathway mediated by VMP1-USP9x-p62, prevents pancreatic cell death. J. Biol. Chem..

[16] Deretic V, Levine B (2009). Autophagy, immunity, and microbial adaptations. Cell Host Microbe.

[17] Benjamin JL, Sumpter R, Levine B, Hooper LV (2013). Intestinal epithelial autophagy is essential for host defense against invasive bacteria. Cell Host Microbe.

[18] Lalazar G (2016). Autophagy confers resistance to lipopolysaccharide-induced mouse hepatocyte injury. Am. J. Physiol. Gastrointest. Liver Physiol..

[19] Gomes LC, Dikic I (2014). Autophagy in antimicrobial immunity. Mol. Cell.

[20] Deretic V (2015). Immunologic manifestations of autophagy. J. Clin. Invest..

[21] Shibutani ST, Saitoh T, Nowag H, Munz C, Yoshimori T (2015). Autophagy and autophagy-related proteins in the immune system. Nat. Immunol..

[22] Jandhyala SM (2017). Altered intestinal microbiota in patients with chronic pancreatitis: implications in diabetes and metabolic abnormalities. Sci. Rep..

[23] Ciocan D (2018). Characterization of intestinal microbiota in alcoholic patients with and without alcoholic hepatitis or chronic alcoholic pancreatitis. Sci. Rep..

[24] Han MM (2019). The alterations of gut microbiota in mice with chronic pancreatitis. Ann. Transl. Med.

[25] Singer M, Deutschman CS, Seymour C (2016). The third international consensus definitions for sepsis and septic shock (sepsis-3). JAMA.

[26] Fleischmann C (2016). Assessment of global incidence and mortality of hospital-treated sepsis. current estimates and limitations. Am. J. Respir. Crit. Care Med.

[27] Russell JA (2006). Management of sepsis. N. Engl. J. Med..

[28] Oberholzer A, Oberholzer C, Moldawer LL (2001). Sepsis syndromes: understanding the role of innate and acquired immunity. Shock.

[29] Fuke, N., Nagata, N., Suganuma, H. & Ota, T. Regulation of gut microbiota and metabolic endotoxemia with dietary factors. *Nutrients* **11**, 10.3390/nu11102277 (2019). 10.3390/nu11102277 PMC6835897 31547555

[30] Gu H (2013). Alcohol exacerbates LPS-induced fibrosis in subclinical acute pancreatitis. Am. J. Pathol..

[31] Gu H (2013). Necro-inflammatory response of pancreatic acinar cells in the pathogenesis of acute alcoholic pancreatitis. Cell Death Dis..

[32] Ammann RW, Heitz PU, Kloppel G (1996). Course of alcoholic chronic pancreatitis: a prospective clinicomorphological long-term study. Gastroenterology.

[33] Ceyhan GO (2007). The neurotrophic factor artemin influences the extent of neural damage and growth in chronic pancreatitis. Gut.

[34] Komatsu M (2005). Impairment of starvation-induced and constitutive autophagy in Atg7-deficient mice. J. Cell Biol..

[35] Rossiter H (2013). Epidermal keratinocytes form a functional skin barrier in the absence of Atg7 dependent autophagy. J. Dermatol. Sci..

[36] Kawaguchi Y (2002). The role of the transcriptional regulator Ptf1a in converting intestinal to pancreatic progenitors. Nat. Genet.

[37] Zhou X (2017). The bile acid receptor FXR attenuates acinar cell autophagy in chronic pancreatitis. Cell death Discov..

[38] Riquelme E (2019). Tumor microbiome diversity and composition influence pancreatic cancer outcomes. Cell.

[39] Yu Y (2016). Progranulin deficiency leads to severe inflammation, lung injury and cell death in a mouse model of endotoxic shock. J. Cell Mol. Med..

[40] Kawasaki T, Kawai T (2014). Toll-like receptor signaling pathways. Front Immunol..

[41] Yamawaki Y, Kimura H, Hosoi T, Ozawa K (2010). MyD88 plays a key role in LPS-induced Stat3 activation in the hypothalamus. Am. J. Physiol. Regul. Integr. Comp. Physiol..

[42] Schmetterer KG, Pickl WF (2017). The IL-10/STAT3 axis: Contributions to immune tolerance by thymus and peripherally derived regulatory T-cells. Eur. J. Immunol..

[43] Bhatia M (2004). Apoptosis versus necrosis in acute pancreatitis. Am. J. Physiol. Gastrointest. Liver Physiol..

[44] Liu Z (2015). Innate immune molecule surfactant protein D attenuates sepsis-induced acute pancreatic injury through modulating apoptosis and NF-kappaB-mediated inflammation. Sci. Rep..

[45] Kaiser AM, Saluja AK, Sengupta A, Saluja M, Steer ML (1995). Relationship between severity, necrosis, and apoptosis in five models of experimental acute pancreatitis. Am. J. Physiol..

[46] Fortunato F (2006). Pancreatic response to endotoxin after chronic alcohol exposure: switch from apoptosis to necrosis?. Am. J. Physiol. Gastrointest. Liver Physiol..

[47] He S, Liang Y, Shao F, Wang X (2011). Toll-like receptors activate programmed necrosis in macrophages through a receptor-interacting kinase-3-mediated pathway. Proc. Natl Acad. Sci. USA.

[48] Newton K (2014). Activity of protein kinase RIPK3 determines whether cells die by necroptosis or apoptosis. Science.

[49] Bode JG, Ehlting C, Haussinger D (2012). The macrophage response towards LPS and its control through the p38(MAPK)-STAT3 axis. Cell Signal.

[50] Brady M, Christmas S, Sutton R, Neoptolemos J, Slavin J (1999). Cytokines and acute pancreatitis. Baillieres Best. Pr. Res Clin. Gastroenterol..

[51] Jakkampudi A (2017). Acinar injury and early cytokine response in human acute biliary pancreatitis. Sci. Rep..

[52] Aguirre A (2014). Defective autophagy impairs ATF3 activity and worsens lung injury during endotoxemia. J. Mol. Med (Berl.).

[53] Zulkefli KL, Houghton FJ, Gosavi P, Gleeson PA (2019). A role for Rab11 in the homeostasis of the endosome-lysosomal pathway. Exp. Cell Res..

[54] Cox D, Lee DJ, Dale BM, Calafat J, Greenberg S (2000). A Rab11-containing rapidly recycling compartment in macrophages that promotes phagocytosis. Proc. Natl Acad. Sci. USA.

[55] Jiang C (2017). Inactivation of Rab11a GTPase in macrophages facilitates phagocytosis of apoptotic neutrophils. J. Immunol..

