# Supplementary material for: Impaired autophagy increases susceptibility to endotoxin-induced chronic pancreatitis
Source: Cell Death Dis. 2020 Oct 21;11(10):889. doi: 10.1038/s41419-020-03050-3 (PMC7578033; doi:10.1038/s41419-020-03050-3)
Supplement: Supplementary file 1 — Supplementary Material [file 41419_2020_3050_MOESM1_ESM.docx]

**Supplemental Material and Methods**.

**Antibodies**

The following antibodies were used for WB: LPS (LSBio, LS-C375096), ATG5 (ABGENT, AP1812a); RIP3 (Abcam, ab62344), MLKL (Abcam, ab194699); α-Amylase (sc-46657), ATG7 (sc-33211), ERK2 (sc-154), Rab5 (sc-46692), Rab7 (sc-376362), Rab11A (sc-166523) (all from Santa Cruz Bio-technology); LC3B (cs-2775), p62 (cs-5114), phosphorylated c-Jun (cs-3270), phosphorylated ERK (cs-9101), phos-phorylated JNK (cs-9255) (all from Cell Signaling Technology). IRDye 680RD goat anti-mouse (926-68070) and IRDye 800CW goat anti-rabbit (926-32211), obtained from LI-COR (Bad Homburg, Germany), were used as secondary antibodies. All other chemicals were from Sigma-Aldrich (Deisenhofen, Germany), if not stated otherwise.

For immunofluorescence (IF) we used the following antibodies: The following primary antibodies were used: murine MLKL (Biorbyt, orb32399); IL-10 (Bioss, bs0698R); NF-κB p65 (Sabbiotech, SAB-11011); Caspase-8 (NB100-56116), Caspase-9 (NB100-56118), IL-6 (NB600-1131) (all from Novus Biologicals); α-Amylase (sc-46657), BAX (sc-526), IL-1β (sc-7884), MCP-1 (sc-28879), phosphorylated STAT3 (sc-8001R), Trypsin (sc-67388) (all from Santa Cruz Biotechnology); Active caspase-3 (ab2302), MPO (ab9535), RIP3 (ab62344), TGF-β (ab66043), TLR4 (ab13556), TNFα (ab66579) (all from Abcam). Secondary anti-mouse (A-31570, SA5-10173)/anti-rabbit (A-31573, SA5-10034) Cy3- or Cy-5 conjugated antibodies were purchased from Thermo Fisher Scientific (Waltham, MA, USA). Secondary anti-rabbit Cy3-, or Cy5-conjugated and anti-mouse Cy3-, or Cy5-conjugated antibodies were purchased from Medac GmbH (Wedel, Germany) and applied for IF.

For immunohistochemistry (IHC) we used anti-E.coli Lipopolysaccharide Core (1:1000 dilution), mAb WN1 222-5 (HM6011-10M), purchased from Hycult biotech (Uden, Netherlands).

**Protein extraction and western blotting**

Immunoblot analysis was performed in order to evaluate variations in the expression of specific proteins involved in apoptosis, autophagy and necrosis signaling. Human and mouse frozen tissues were homogenized on ice as described previously.(Fortunato, Berger et al. 2007, Fortunato, Burgers et al. 2009) Protein loading control was performed with ERK2 for mice and GAPDH for human, after using a restore western blot stripping buffer plus (Pierce Biotech., Rockford, US) to ensure equal protein loading, according to the instructions. Protein concentrations were determined by BCA Protein Assay Kit (Thermo Fisher Scientific, Waltham, MA, USA) according to the manufacturer’s instructions. Proteins were separated using SDS-polyacrylamide gels and transferred from the gel to nitrocellulose membranes. Membranes were exposed to the primary antibodies, followed by the staining with the secondary antibodies. Protein fragments were scanned with the ODYSSEY® CLx (LI-COR) imaging systems, further processed and analyzed by using computer-assisted software ImageJ (NIH, Bethesda, MD, USA).

**Supplementary Figure 1. (A)** Total pancreas and liver protein from Atg7^F/F^ and Atg7^Δpan^ mice immunoblotted with the antibodies ATG7, p62, ATG5, LC3-Ι and LC3-II were shown as the representative SDS-PAGE autoradiograph with Erk1/2 serving as a loading control. **(B)** Decreased body weight in 12 weeks old Atg7^Δpan^ mice. Data were plotted as means ± SEM for the numbers of animals in each group as indicated in the graph. **(C)** Loss of body weight in Atg7^F/F^ and Atg7^Δpan^ mice after LPS treatment within 24 hours were plotted as means ± SEM for each group (n = 5). **(D)** Pancreatic α-Amylase was determined by WB analysis using the ratio of α-Amylase and ERK1/2, indicated by the representative SDS-PAGE autoradiograph. Ratios were plotted as means ± SEM, n = 4 for each group. **p* < 0.05, ***p* < 0.01, ****p* < 0.001.

**Supplementary Figure 2. (A)** Serum α-amylase and **(B)** serum lipase **(C)** serum glucose and **(D)** serum lactate dehydrogenase (LDH) in Atg7^F/F^ and Atg7^Δpan^ mice with and without LPS (n= 4-6). All values were plotted as means ± SEM for 4 to 7 animal serum samples.

**Supplementary Figure 3. (A)** Representative IF images stained for DAPI (blue) and Bax (green), as well as representative FACS-like scattergrams quantification of Bax and DAPI expression in Atg7^F/F^ and Atg7^Δpan^ mice. **(B)** Representative IF colocalization images stained for DAPI (blue), α-Amylase (red) and Caspase-3 (green), as well as representative FACS-like scattergrams quantification of Caspase-3 and α-Amylase expression in Atg7^F/F^ and Atg7^Δpan^ mice. **(C)** Representative IF colocalization images stained for DAPI (blue), α-Amylase (red) and Caspase-8 (green), as well as representative FACS-like scattergrams quantification of Caspase-8 and α-Amylase expression in Atg7^F/F^ and Atg7^Δpan^ mice. **(D)** Representative IF colocalization images stained for DAPI (blue), α-Amylase (red) and Caspase-9 (green), as well as representative FACS-like scattergrams quantification of Caspase-9 and α-Amylase expression in Atg7^F/F^ and Atg7^Δpan^ mice. (20x objective; Scale bar = 50 µm).

**Supplementary Figure 4. (A)** Representative WB image of RIP3 and ERK1/2 (loading control) and the ratio of RIP3 to ERK1/2 were plotted as means ± SEM of 4 animals per group. **(B)** Representative WB image of MLKL with ERK1/2 (loading control) and the ratio of MLKL to ERK1/2 were plotted as means ± SEM of 4 animals per group. **(C)** Representative IF colocalization images stained for DAPI (blue), α-Amylase (red) and IL-10 (green), as well as representative FACS-like scattergrams quantification of IL-10 and α-Amylase expression in Atg7^F/F^ and Atg7^Δpan^ mice. **(D)** Representative IF colocalization images stained for DAPI (blue), α-Amylase (red) and TGF-β (green), as well as representative FACS-like scattergrams quantification of TGF-β and α-Amylase expression in Atg7^F/F^ and Atg7^Δpan^ mice. **(E)** Expression profile of IL-10 and TGF-β in acinar cells were plotted as means ± SEM of 6 animals per group. (20x objective; Scale bar = 50 µm) **p* < 0.05, ***p* < 0.01, ****p* < 0.001, *****p* < 0.0001.

**Supplementary Figure 5. (A)** Representative WB image of p-JNK, p-ERK, p-c-Jun and ERK1/2 (loading control). **(B)** Expression of p-JNK, p-ERK, p-c-Jun to the ratio of ERK1/2 were plotted as means ± SEM of 4 animals per group. **p* < 0.05, ***p* < 0.01, ****p* < 0.001, *****p* < 0.0001.

**Supplementary Figure 6.** **(A)** Representative H&E staining of pancreatic tissue images from Lamp2^+/+^ and Lamp2^y/-^ mice tissue sections with and without LPS (White arrow indicate strong acinar vacuolization (20 x objective; Scale bar = 50 μm). **(B)** Histopathological evaluation for pancreatic injury, examined for edema and acinar cell vacuolization, 24 h after LPS injection in Lamp2^+/+^ and Lamp2^y/-^ mice. The scores were plotted as means ± SEM for each group (n = 5). **(C)** Decreased body weight in 25 weeks old Lamp2^y/-^ mice. Data were plotted as means ± SEM for the numbers of animals in each group as indicated in the graph. **(D)** Serum α-amylase and serum lipase and **(E)** serum glucose and serum lactate dehydrogenase (LDH) in Lamp2^+/+^ and Lamp2^y/-^ mice with and without LPS (n= 4-6). All values were plotted as means ± SEM for 4 to 5 animal serum samples. **p* < 0.05, ***p* < 0.01, ****p* < 0.001, *****p* < 0.0001.

**Supplementary Figure 7.** **(A)** Pancreatic expression of TLR4 in acinar cells of Lamp2^+/+^ and Lamp2^y/-^ mice, 3 and 24 hours after LPS were determined by IF FACS-like quantitation and plotted as means ± SEM (n= 5 per group). **(B)** Pancreatic expression of p65 in acinar cells of Lamp2^+/+^ and Lamp2^y/-^ mice, 3 and 24 hours after LPS were determined by IF FACS-like quantitation and plotted as means ± SEM (n= 5 per group). **(C)** Pancreatic expression of α-amylase in acinar cells of Lamp2^+/+^ and Lamp2^y/-^ mice, 3 and 24 hours after LPS were determined by IF FACS-like quantitation and plotted as means ± SEM (n= 5 per group). **p* < 0.05, ***p* < 0.01, ****p* < 0.001, *****p* < 0.0001.

**Supplementary Figure 8.** **(A)** Representative IF colocalization images stained for DAPI (blue), α-Amylase (red) and RIP3 (green), as well as representative FACS-like scattergrams quantification colocalization of RIP3 and α-Amylase expression in Lamp2^+/+^ and Lamp2^y/-^ mice 24 hours after LPS. **(B)** Pancreatic expression levels of RIP3 in acinar cells from Lamp2^+/+^ and Lamp2^y/-^ mice, 24 hours after LPS. Values were determined by FACS-like IF quantitation and plotted as means ± SEM. **(C)** Representative IF colocalization images stained for DAPI (blue), α-Amylase (red) and MLKL (green), as well as representative FACS-like scattergrams quantification colocalization of MLKL and α-Amylase expression of Lamp2^+/+^ and Lamp2^y/-^, mice 24 hours after LPS. **(D)** Pancreatic expression levels of MLKL in acinar cells in Lamp2^+/+^ and Lamp2^y/-^ mice 24 hours after LPS, Values were determined by FACS-like IF quantitation and plotted as means ± SEM. **p* < 0.05.

**Supplementary Figure 9.** **(A)** Pancreatic expression of TNFα in acinar cells of Lamp2^+/+^ and Lamp2^y/-^ mice, 3 and 24 hours after LPS were determined by IF FACS-like quantitation and plotted as means ± SEM (n= 4 to 5 animals per group). **(B)** Pancreatic expression of IL-1β in acinar cells of Lamp2^+/+^ and Lamp2^y/-^ mice, 3 and 24 hours after LPS were determined by IF FACS-like quantitation and plotted as means ± SEM (n= 4 to 5 animals per group). **(C)** Pancreatic expression of MCP-1 in acinar cells of Lamp2^+/+^ and Lamp2^y/-^ mice, 3 and 24 hours after LPS were determined by IF FACS-like quantitation and plotted as means ± SEM (n= 4 to 5 animals per group). **p* < 0.05, ***p* < 0.01, ****p* < 0.001, *****p* < 0.0001.

Fortunato, F., I. Berger, M. L. Gross, P. Rieger, M. W. Buechler and J. Werner (2007). "Immune-compromised state in the rat pancreas after chronic alcohol exposure: the role of peroxisome proliferator-activated receptor gamma." J Pathol **213**(4): 441-452.

Fortunato, F., H. Burgers, F. Bergmann, P. Rieger, M. W. Buchler, G. Kroemer and J. Werner (2009). "Impaired autolysosome formation correlates with Lamp-2 depletion: role of apoptosis, autophagy, and necrosis in pancreatitis." Gastroenterology **137**(1): 350-360, 360 e351-355.
