# Supplementary figures and images for: Impaired autophagy increases susceptibility to endotoxin-induced chronic pancreatitis
Source: Cell Death Dis. 2020 Oct 21;11(10):889. doi: 10.1038/s41419-020-03050-3 (PMC7578033; doi:10.1038/s41419-020-03050-3)

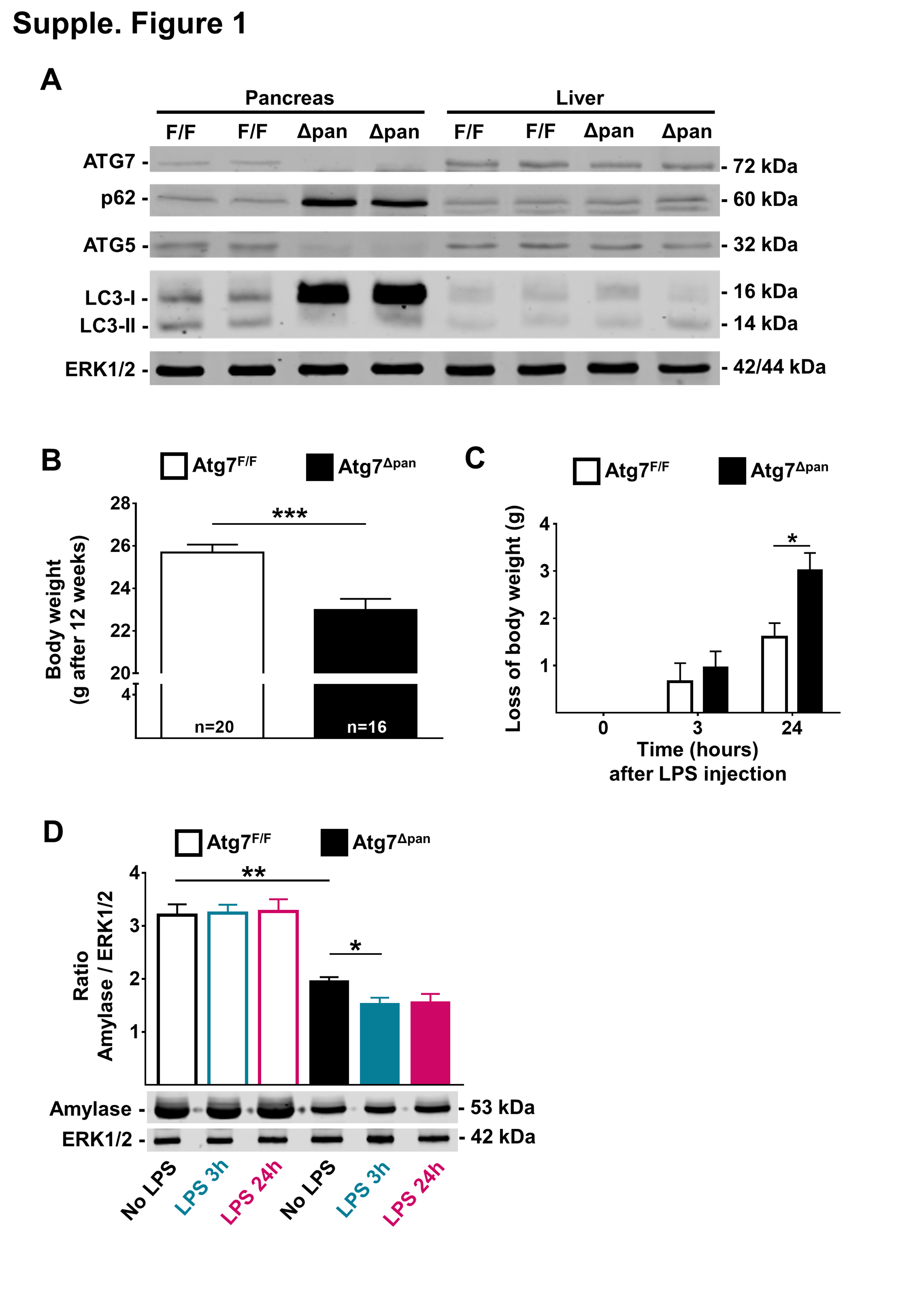

Supplement: Supplementary file 2 — Supplementary Fig. 1 [file 41419_2020_3050_MOESM2_ESM.tif]

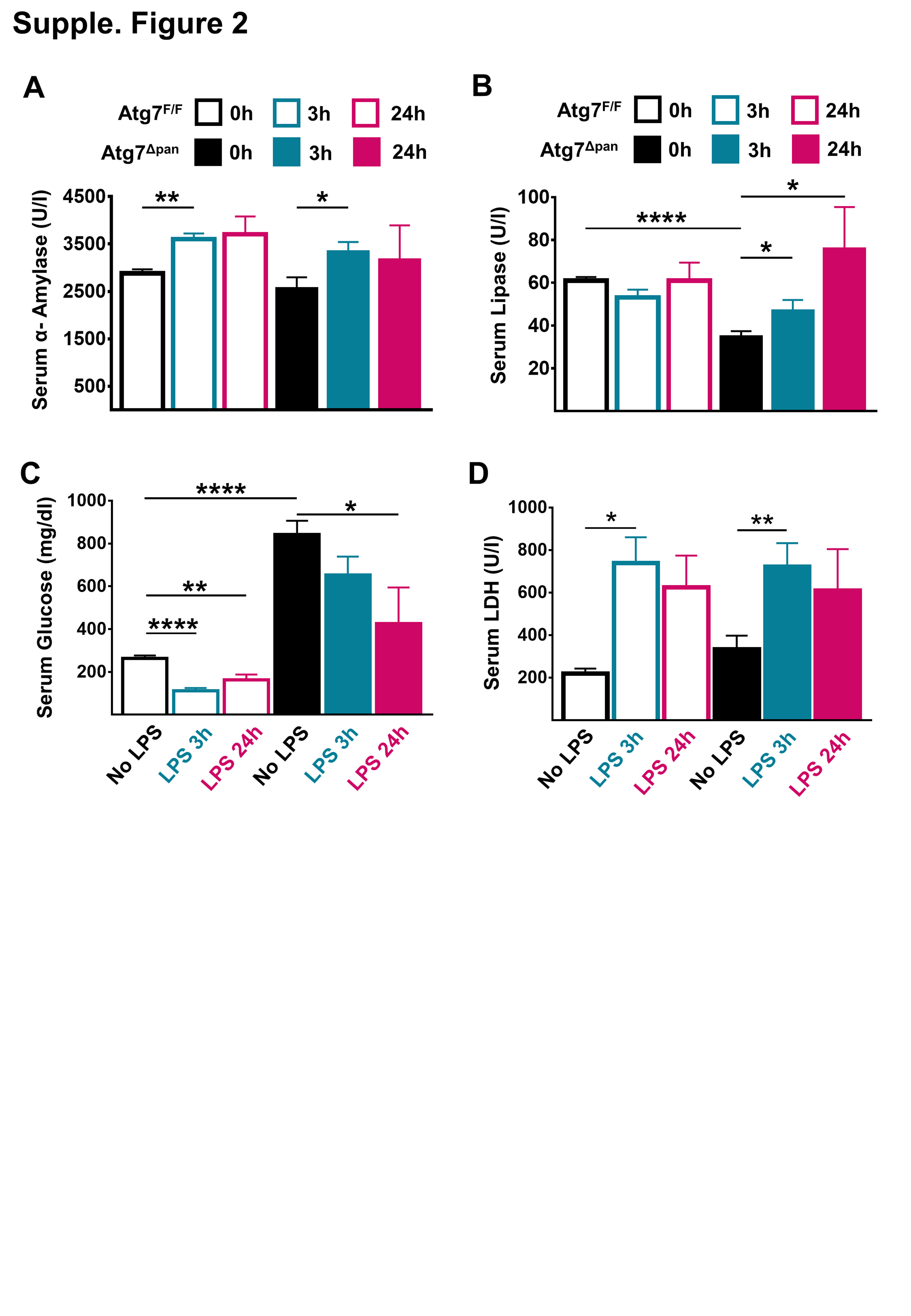

Supplement: Supplementary file 3 — Supplementary Fig. 2 [file 41419_2020_3050_MOESM3_ESM.tif]

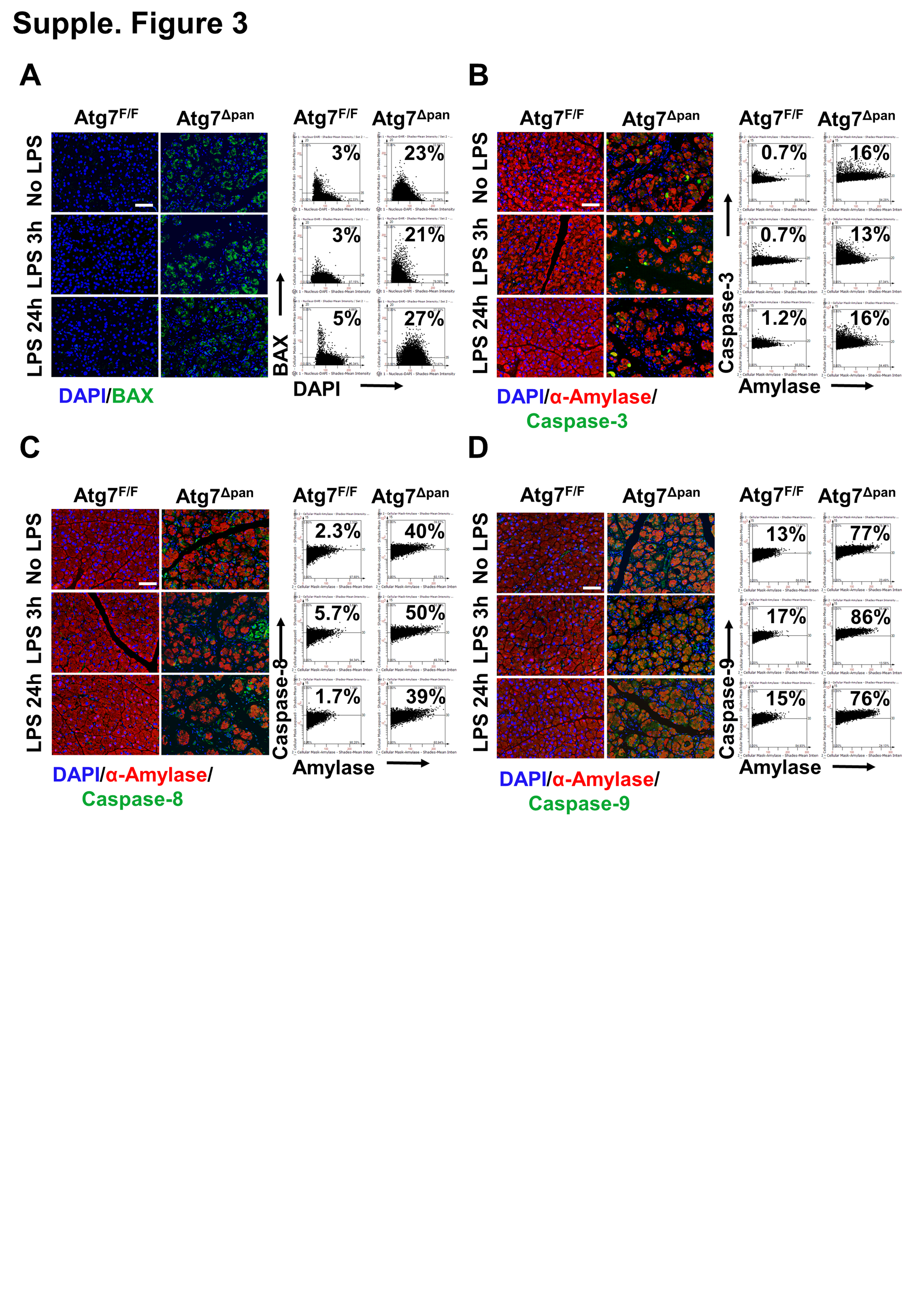

Supplement: Supplementary file 4 — Supplementary Fig. 3 [file 41419_2020_3050_MOESM4_ESM.tif]

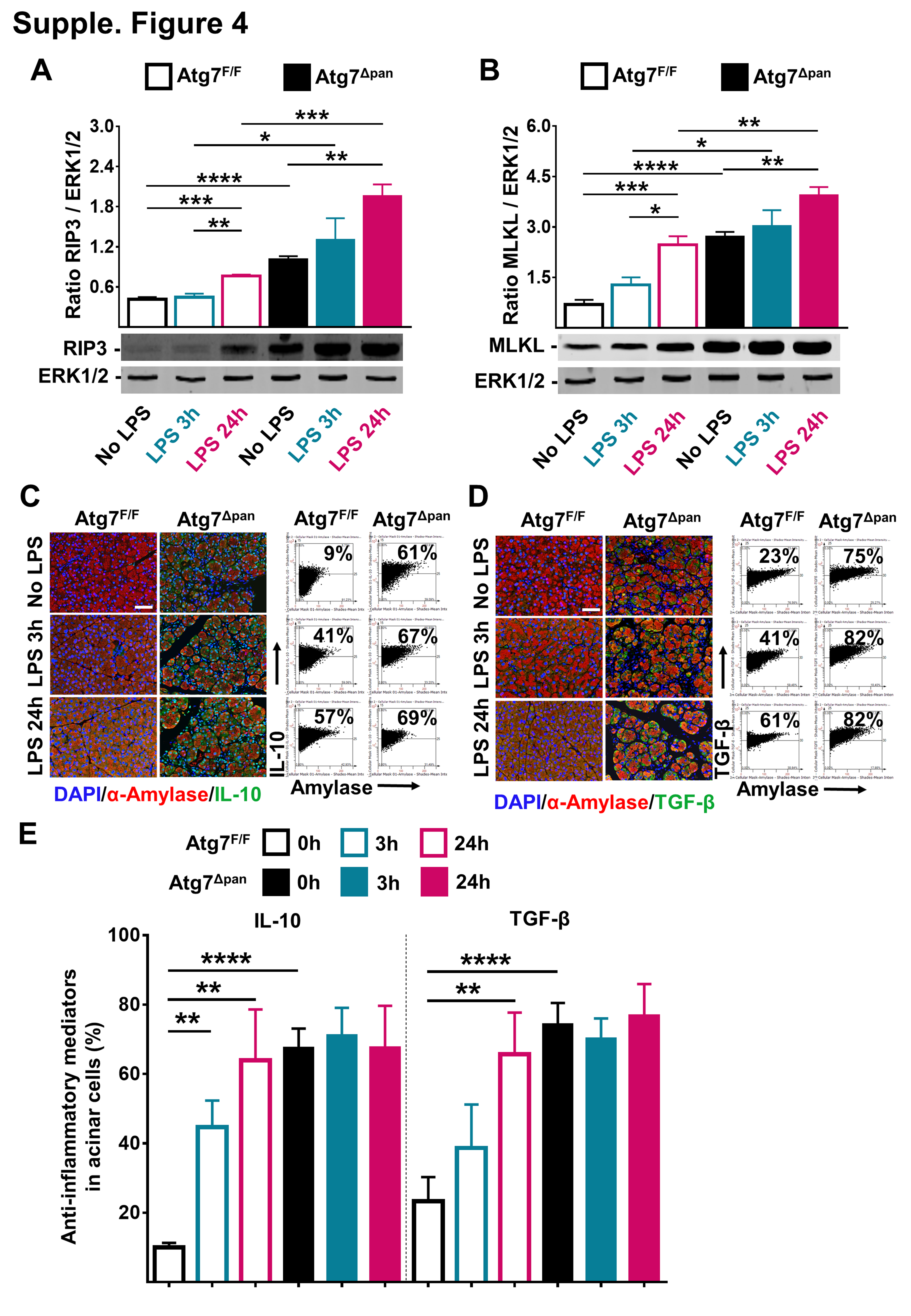

Supplement: Supplementary file 5 — Supplementary Fig. 4 [file 41419_2020_3050_MOESM5_ESM.tif]

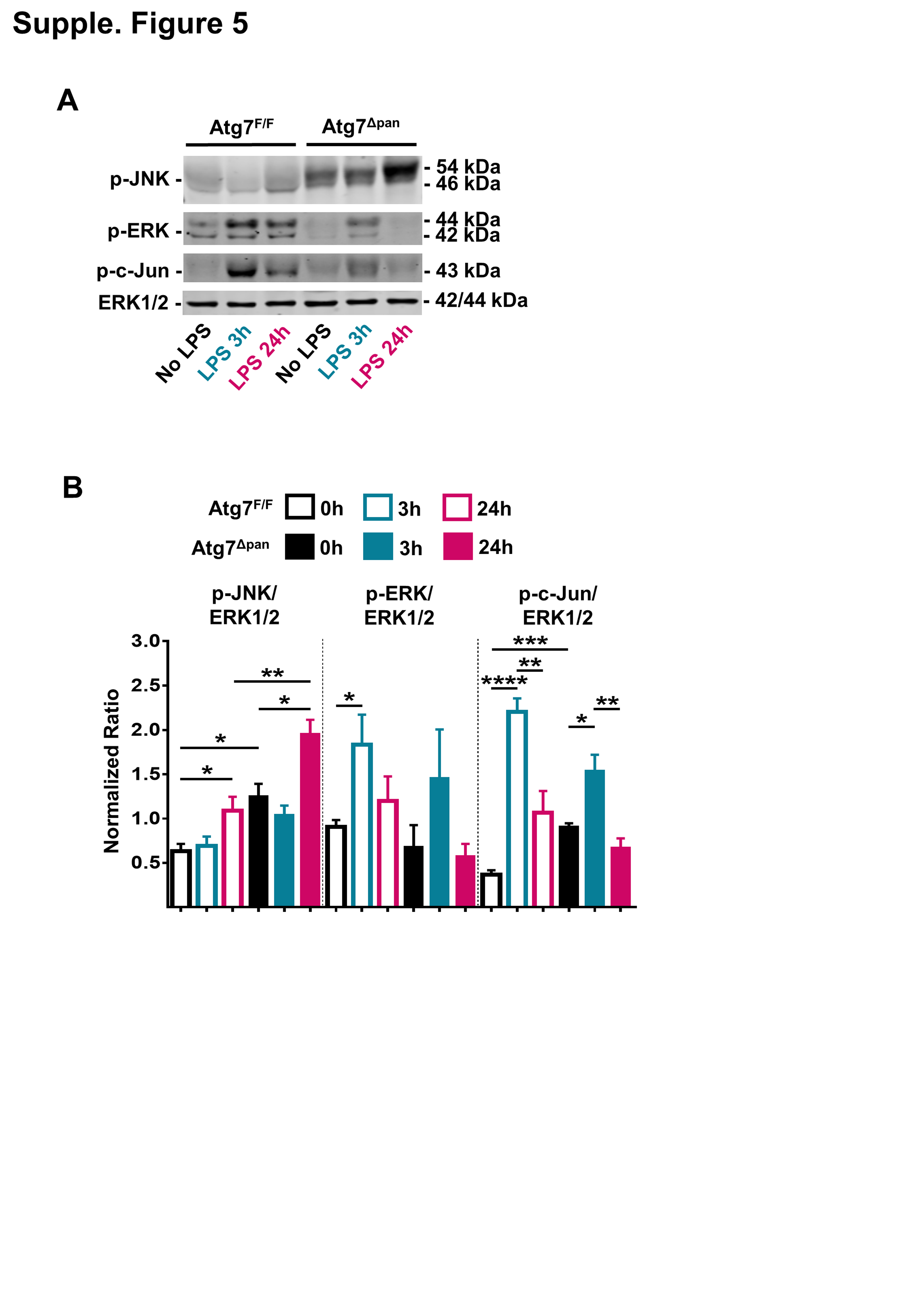

Supplement: Supplementary file 6 — Supplementary Fig. 5 [file 41419_2020_3050_MOESM6_ESM.tif]

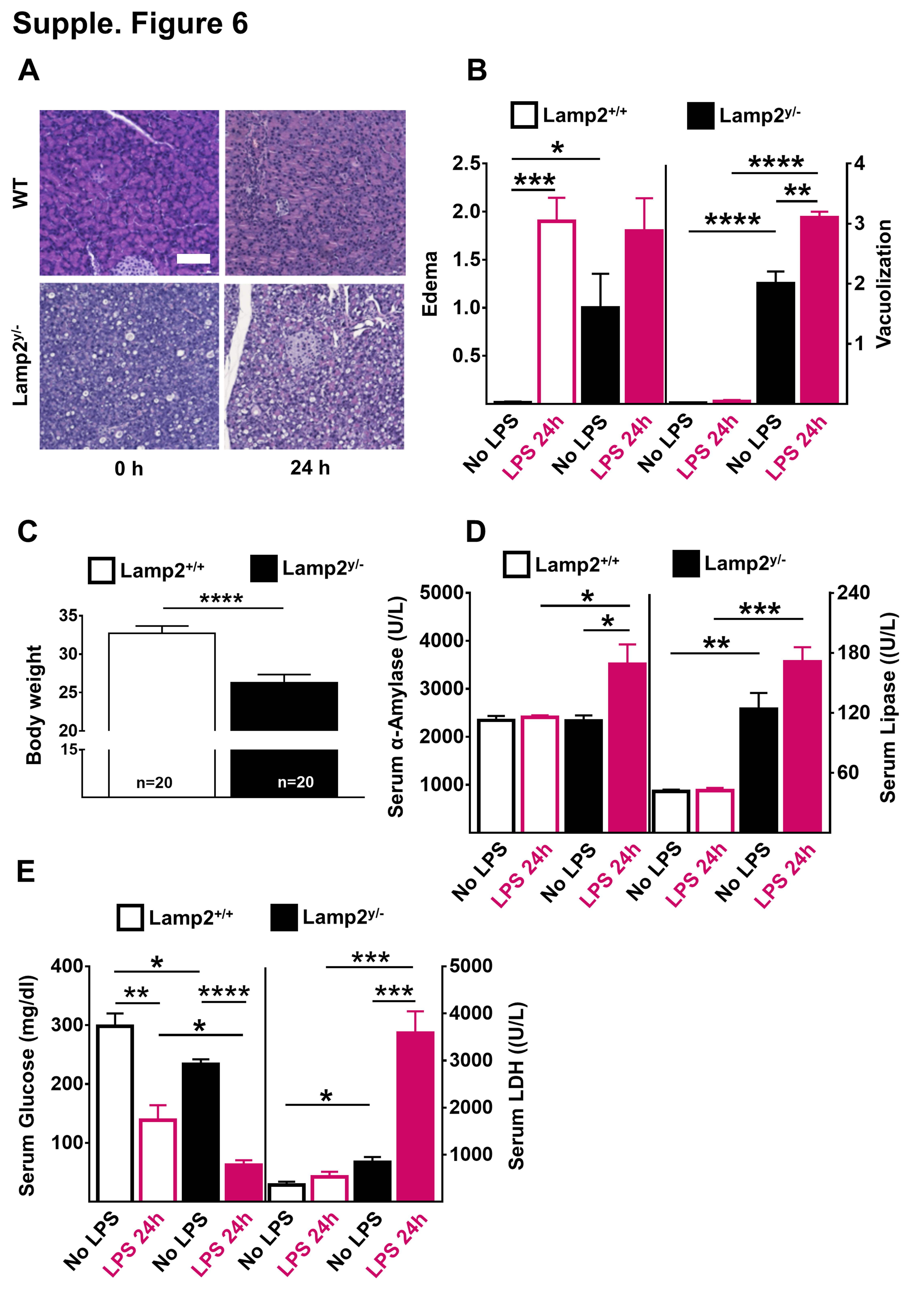

Supplement: Supplementary file 7 — Supplementary Fig. 6 [file 41419_2020_3050_MOESM7_ESM.tif]

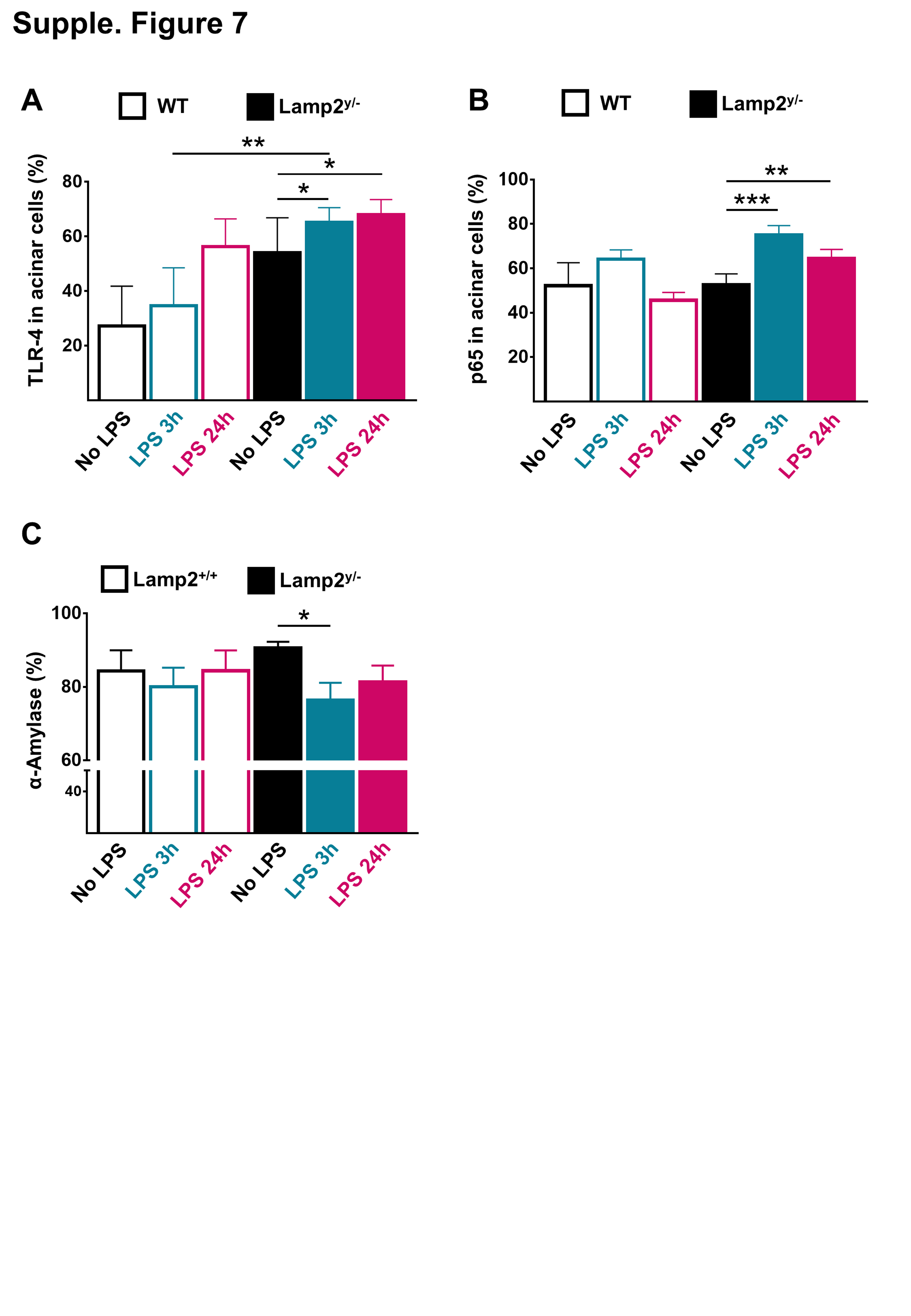

Supplement: Supplementary file 8 — Supplementary Fig. 7 [file 41419_2020_3050_MOESM8_ESM.tif]

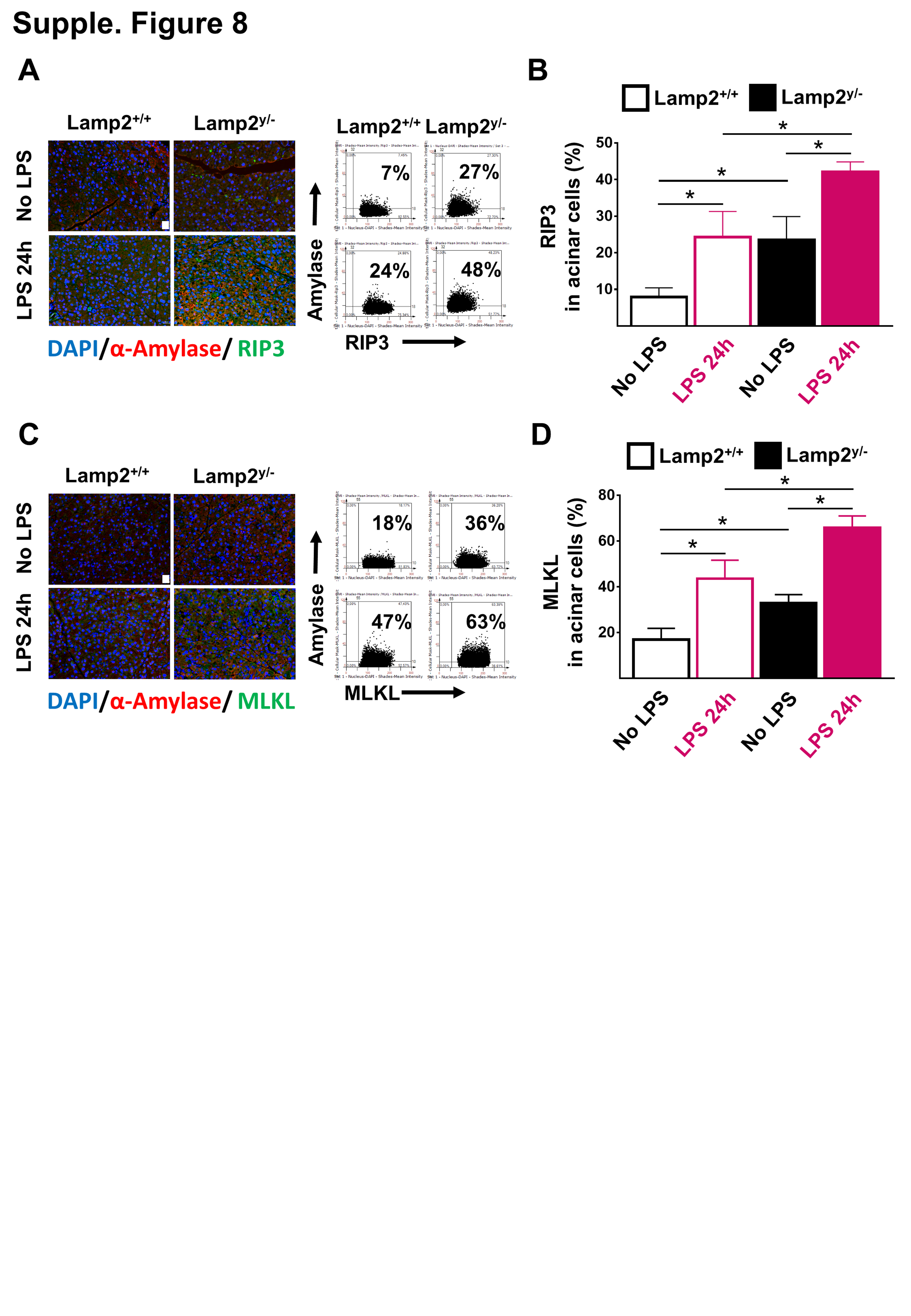

Supplement: Supplementary file 9 — Supplementary Fig. 8 [file 41419_2020_3050_MOESM9_ESM.tif]

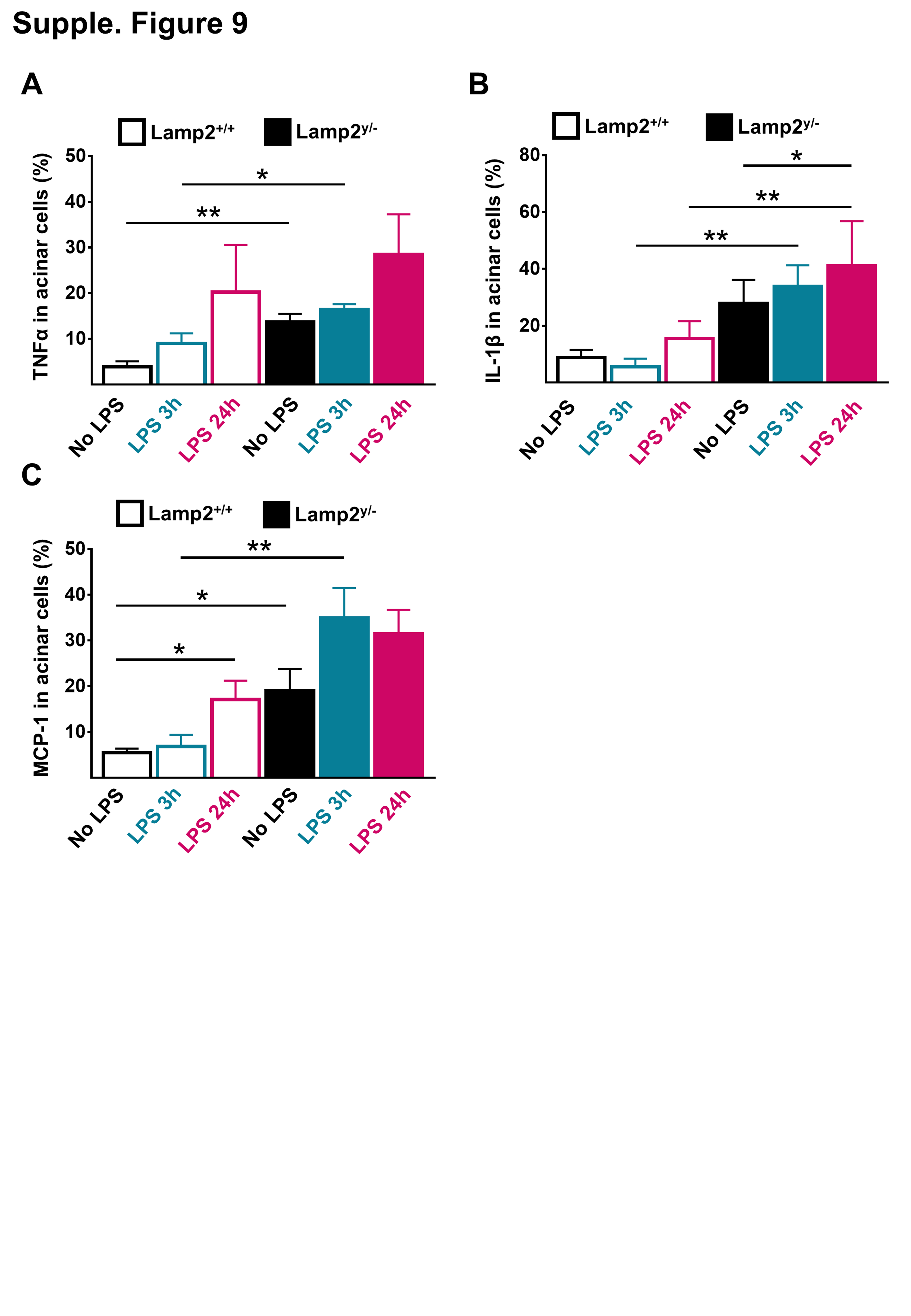

Supplement: Supplementary file 10 — Supplementary Fig. 9 [file 41419_2020_3050_MOESM10_ESM.tif]
